# Clinical research on homeopathic preparations: protocol template for a series of systematic reviews

**DOI:** 10.1186/s13643-026-03168-z

**Published:** 2026-04-07

**Authors:** Martin Loef, Robbert van Haselen, Stephan Baumgartner

**Affiliations:** 1 Institute of Integrative Medicine, University of Witten/Herdecke, Witten, Germany; 2 Society for Clinical Research, Berlin, Germany; 3 International Institute for Integrated Medicine, London, UK; 4 Institute of Complementary and Integrative Medicine, University of Bern, Bern, Switzerland

**Keywords:** Homeopathy, Systematic review, Efficacy, Effectiveness, Safety

## Abstract

**Background:**

The clinical benefits of homeopathic preparations (HPs) used in anthroposophic medicine and homeopathy remain a topic of debate. Systematic reviews (SRs), with or without meta-analyses (MAs), that assess the literature in line with scientific standards and account for the complex nature of these interventions are lacking for many health conditions. This project aims to conduct SRs to evaluate the efficacy, effectiveness, and safety of HPs for selected patient populations, using a pluralistic approach that considers internal and model validity.

**Methods:**

This protocol follows PRISMA-P. A comprehensive literature search will include the HOMIS database, MEDLINE, EMBASE, CINAHL, Cochrane Central Register of Controlled Trials, AMED, LILACS, topic-specific sources, citation indices, trial registers, and grey literature, including preprint servers. Eligible studies will have a prospective, longitudinal design and include randomized controlled trials and non-randomized comparative studies published in various languages, evaluating HP interventions for efficacy, effectiveness, and/or safety in health conditions preselected through expert consensus. All patient types, interventions, co-interventions, therapeutic goals, and comparison groups will be eligible. Research questions and outcomes will be developed with consideration of the patient perspective.

Each SR will be conducted by a multidisciplinary team, supported by an advisory group. Risk of bias will be assessed using ROB-2 and ROBINS-I. Model validity and the efficacy–effectiveness spectrum will be evaluated using MVHT and RITES. Intervention complexity will be analyzed with an adapted iCAT_SR. Where meta-analysis is not feasible, a narrative synthesis following SWiM will be conducted; otherwise, quantitative synthesis will be performed. Certainty of evidence will be assessed using GRADE.

**Discussion:**

This series of SRs and MAs will investigate the effects of HPs as complex interventions in healthcare and aims to establish an evidence base regarding their efficacy, effectiveness, and safety. By integrating assessments of internal and model validity and potential effect modifiers, the reviews aim to provide a more comprehensive understanding of the conditions under which HPs may yield clinical benefits. In addition, the project may identify research gaps and methodological shortcomings to inform future studies.

**Systematic review registration:**

PROSPERO CRD420251017029.

**Supplementary Information:**

The online version contains supplementary material available at 10.1186/s13643-026-03168-z.

## Background

### Description of the intervention

Homeopathy (Greek *homoios* = same or similar, *pathos* = suffering) is a medical system based on the “Law of Similars” or the principle of “like cures like”. This idea holds that a substance that can induce symptoms in a healthy individual may also alleviate similar symptoms in a patient suffering from an illness [1, 2]. Homeopathy was developed in the late eighteenth century by the German physician Samuel Hahnemann (1755–1843) [3].

For therapeutic purposes, the substances chosen according to the simile principle to treat patients are not applied in crude raw form, but pharmaceutically processed as “Homeopathic preparation” (HP).^1^ HPs are prepared according to the regulations of current pharmacopoeias (e.g. the European Pharmacopoeia, or the German, US, Indian, or Brazilian Homeopathic Pharmacopoeia) and can be made from a variety of organic and inorganic substances which are stepwise diluted in water, alcohol, or other carrier substances. This process, known as potentization, involves serial dilutions, usually in steps of 1:10 or 1:100, with succussion (vigorous shaking) between each dilution step [2].

The homeopathic treatment approach in its purest, original form markedly diverges from conventional medicine, emphasizing highly personalized care where practitioners prescribe HPs based on a patient’s unique pattern of symptoms rather than solely on the diagnosed disease. In this so-called individualized, or “classical” homeopathy, practitioners analyze a broad range of symptoms - including mental, emotional, and physical aspects - and recommend a single HP that aligns closely with the patient’s symptom profile. In practice, however, several types of homeopathy are used, including non-individualized approaches [2].

In contrast, non-individualized homeopathy, also known as pluralistic homeopathy, applies multi-constituent HPs and homeopathic interventions with more than one single HP at a time, mostly combing different remedies suitable for a given conventional diagnosis. Other related forms of therapy exist, such as isopathy, which is the use of homeopathic dilutions of allergens or toxic or causative infectious agents. Anthroposophic Medicine also applies HPs, in most cases either single remedies in the sense of clinical homeopathy for particular clinical indications, or as complex remedies.

### Conditions and setting

The use of HPs has been studied across a broad spectrum of healthcare settings, including primary care and family practices, private practices, hospitals and specialized clinics, in different cultures (e.g. [4–6]). It is utilized for both acute and chronic conditions (e.g. infectious diseases, pain management, mental health, and complementary cancer care [7]), serving preventive as well as therapeutic purposes.

### Prevalence of usage

The popularity of homeopathy and consequently HPs has persisted over two centuries, with significant usage documented in both Western and Asian nations. A systematic review of worldwide surveys on homeopathy use found that the 12-month prevalence of homeopathy usage—whether through treatment by homeopaths or over-the-counter-HPs—ranged from 0.7% to 9.8%, with a median of 3.9% [8]. The highest use was reported by a survey in Switzerland, possibly due to the inclusion of homeopathy in public health insurance. In India, homeopathy is widespread and is seen as one of the traditional, complementary and/or alternative systems of medicine in the context of AYUSH (Ayurveda, Yoga and Naturopathy, Unani, Siddha and Homeopathy) [6]. According to data from the producers group ECHAMP (European Coalition of Homeopathic and Anthroposophic Medical Products) there is a widespread use of HP in France, Germany, Italy, Spain, and Austria [9]. In the same vein, a survey commissioned by the “Deutsche Homöopathie Union” found that 54% of the participants (*n* = 2006 people in Germany, age 18 +) have had experience with homeopathy and a further 16% would consider using it [10].

### How the intervention might work

Treatments in this series of SRs will include HPs applied in individualized and non-individualized form, i.e. homeopathic remedies, whether as single or multiple components, initiated by homeopaths, healthcare providers or patients themselves. The remedies may have been applied with preventive or therapeutic scope.

Originally, Hahnemann attributed the medical effect of HPs to the stimulation of an organizing principle inherent in the body. In the meantime, further theories have been formulated which attempt to align explanations of the mode of action of HPs with modern science (see [11] for an overview). These are partially supported by physico-chemical investigations, which, however, require further validation or additional testing to allow firmer conclusions to be drawn [12–14].

In addition, homeopathy is a whole medical system that addresses the disease on various levels, leading to intervention complexity, when therapy is tailored to the individual patient and when different HPs are used in the course of the therapy, due to the training, skills, and experience of the therapist, as well as the patient’s medical history, experiences, and attitudes, alongside cultural and interpersonal factors that may also play a role. For example, the dynamic interactions and close relationship between patients and providers including empathy, in-depth anamnesis of bodily and psychological complaints, the remedy matching process and the remedies themselves were identified as possible active elements of homeopathy [15].

Therefore, SRs of homeopathy face unique challenges that may lead to inadequate conclusions if its complex nature and real-word evidence are not taken into account [16].

### Why it is important to do these reviews

In contrast to other areas of complementary medicine (e.g. mistletoe therapy [17–20]), the status of comparative clinical research on homeopathic medicines has not been systematically analyzed for distinct patient populations. Although, as for the health sciences in general [21], there is a plethora of redundant and low-quality reviews, there is a lack of reliable evidence that meets contemporary scientific standards. A selection of published systematic reviews on homeopathy for the treatment or prevention of human diseases is shown in Tables 1 and 2 for all and specific patient populations, respectively. The number of medical conditions is limited and the assessments of risk of bias, model and external validity, the inclusion of non-randomized studies and the certainty of evidence are only partially implemented.

**Table 1:** Systematic reviews on comparative studies with a single focus on homeopathy for the treatment or prevention of human diseases without a limitation on indications with a registered protocol and published 2017 or later

Reference	Patient; intervention; control; outcome	Study types	Meta-analysis	ROB	MV	EV	GRADE
[22]	Any indication; IH, non-IH, any HO; placebo; effect estimate of MA of high-quality trials	SR		x			x
[23]	Any indication; any HO; conventional medicines, usual care, waiting lists, other CAM; AE, adverse drug reactions, tolerability, safety, homeopathic aggravations	NRSI	x				
[24]	Any indication, non-IH; other than placebo; any outcome	RCT	x	x	x	x	
[25]	Any indication; IH; other than placebo; any outcome	RCT	x	x	x	x	
[26]	Any indication; non-IH; placebo; any outcome	RCT	x	x	x		

Literature search: On January, 23rd, 2025, we searched Epistemonikos and Pubmed (advanced search) with the terms (systematic review[Title/Abstract] OR meta-analysis[Title/Abstract]) AND (homeopathy[Title/Abstract] OR homoeopath*[Title/Abstract] OR homeopath*[Title/Abstract]). No filters were applied. In addition, we searched google scholar for “homeopathy systematic review”. The selection was based on the criteria in the title of the table, retracted publications are not shown. *AE* adverse events, *EV* external validity, *GRADE* Grading of Recommendations, Assessment, Development and Evaluation, *HP* homeopathic preparation, *HO* homeopathy, *IH* individualized homeopathy, *MA* meta-analysis, *MV* model validity, *NRSI* non-randomized study on intervention, *non-IH* non-individualized homeopathy, non-randomized studies of interventions, *RCT* randomized controlled trial, *ROB* risk of bias, *SR* systematic review

**Table 2:** Indication-specific systematic reviews on comparative studies with a single focus on homeopathy for the treatment or prevention of human diseases with a registered protocol and published 2017 or later

Reference	Patient; intervention; control; outcome	Study types	Meta-analysis	ROB	MV	EV	GRADE
[27]	Otitis media; IH, non-IH; any control; symptoms, use of antibiotics, others	RCT, NRSI	x	x			x
[28]	Acute respiratory tract infection in children; oral HP (simplex, complex, IH, non-IH); placebo or conventional; cure, severity, AE, use of antibiotics, other	RCT	x	x			x
[29]	Urological disorders; any HO; any control and uncontrolled; no outcome defined	RCT, NRSI		x			
[30]	Postoperative recovery; *Arnica montana;* placebo, active; any outcome	RCT, NRSI	x	x			
[31]	Major depressive disorder, generalized anxiety disorder, attention-deficit/hyperactivity disorder, and premenstrual syndrome/dysphoric disorder; any HO (add-on or mono); placebo, usual care; severity of psychiatric disorders	RCT	x	x			
[32]	Irritable bowel syndrome; any HO; placebo, no treatment, active control; symptoms, AE, others	RCT, NRSI	x	x			x
[33]	Chronic bronchial asthma; any HO; placebo, conventional and uncontrolled; any outcome	RCT, NRSI		x			
[34]	Diabetes and obesity; any HO; placebo, any active drug; HbA1c, body mass index	RCT					
[35]	Allergic rhinitis; any HP, placebo, conventional, other HP; symptoms, AE, others	RCT	x	x			x

Abbreviations: see Table 1

A systematic review (SR) with meta-analyses of placebo-controlled randomized efficacy trials of homeopathy reports positive effects for homeopathy versus placebo in five out of six meta-analyses, but conflicting results regarding the potential risk of bias [22]. However, the included meta-analyses and the studies in Table 1 were not conducted with specific patient populations, did not include all available evidence according to the latest bibliography on clinical research with homeopathic preparations [36], and only included randomized controlled trials (RCTs).

In addition, 27 other systematic reviews are planned that investigate homeopathy in human medical conditions and have been registered as protocols (as of May 2025; the search will be repeated quarterly to avoid redundant SRs [37], see supplement S.1 for details).

A recent study by Herman et al. (2024) reviewed 99 studies on homeopathy regarding their internal, external, and model validity. These authors convened an expert panel to identify research gaps and concluded the need to adhere to standard and homeopathy-specific reporting guidelines, to detect exemplar studies and to increase homeopathic research expertise [38].

Most SRs to date do not comprehensively include all the available literature, fail to consider quality of reporting, model validity or generalizability of the underlying studies, make no attempt to address the complexity of the intervention in analysis and discussion, and rarely assess the certainty of evidence. The perspectives of the general practitioner and/or homeopath are neglected, as are those of the patient, and their relationship with the clinician. The contextualization of homeopathy as an individualized, patient-centered therapy is missing, as is the consideration of evidence from non-experimental studies [39, 40].

### Challenges of homeopathic evidence research

Homeopathy is controversial and has been a focus of debate throughout its history [2]. It has been described as “implausible” [41] or as a “null field” [42] mainly due to the lack of a “credible” mechanism of action of highly diluted HPs [43].

Corresponding clinical research is challenging due to the complexity of the intervention, for instance, the standardization of a therapy in RCTs conflicts with the individualized nature of the therapy and its reliance on the patient-therapist relationship. Furthermore, there may be few studies on homeopathy available for different indications, and some may exhibit incomplete reporting, have small sample sizes, methodological weaknesses and a high degree of heterogeneity with regard to patients, interventions, controls, and outcome parameters. It has been speculated that the validity of the body of evidence may be affected by publication bias, which could lead to an overestimation of the true effect size of HPs [44]. Additional influences that are discussed include conflicts of interest and non-neutral reporting [45].

### Rationale and scope

The overarching goal of the present review protocol is to evaluate the evidence regarding the effectiveness and safety of HPs in specific populations and outcomes, and in accordance with current guidelines [46] and framing homeopathy as a complex, system-orientated. Moreover, the SRs will account for the unique characteristics of the intervention, which is in contrast to mono-therapeutic drug studies. This is achieved through a comprehensive literature search, the inclusion of grey literature and NRSIs, extraction of multiple outcomes and possible effect moderators such as settings and other contextual factors, homeopathy-specific assessment tools, accounting for the complexity of the intervention through complementary methods and outcome measures, incorporation of the clinical perspective by involving homeopaths in all steps of the SR and consideration of patient perspectives and the environment in which homeopathy is applied, using a broad and a non-contextualized approach regarding the meta-analyses and the certainty of evidence assessment, respectively.

The individual SRs will be placed within the common framework of a discussion that aims to highlight the possibilities and limitations of evidence synthesis with a particular focus on individualized and holistic interventions [47]. The evidence synthesis is based on the theoretical and methodological groundwork of various groups, covering:

- Theories [11] of and basic research [12] on homeopathy.
- The MVHT and RITES to assess model validity and pragmatism respectively (see chapter 6.7).
- Recommendations for summarizing evidence from homeopathic intervention studies (Sum-HomIS) [48].
- A preliminary investigation for assessing and selecting the indication-related topics to be addressed.

The patient populations to be examined were pre-selected by two consecutive expert consensus panels held in October and November 2023. The panels included participants specialized in research methodology, homeopathy, evidence synthesis, anthroposophy, and basic research. The HOMIS database was used as the data basis for the selection process and includes prospective, randomized, and non-randomized studies comparing HPs with one or more controls [36]. In June 2023, the HOMIS database was searched for ICD-10 categories with four or more studies as well as for the 10 single and complex homeopathic remedies with the highest number of publications. The selection was based on.

1. The number of available studies per patient population. We selected clinical populations if there were *n* ≥ 7 studies for RCTs and NRSIs combined. Our rationale behind this criterion was to include patient populations with numerous studies, enabling the examination of intervention complexity through study comparisons and joint analyses.
2. Exclusion of HPs from a single company.
3. The exclusion of patient populations for which a SR on homeopathy had recently been published and/or was ongoing (as assessed by literature search in PubMed and PROSPERO, respectively).

The list of pre-selected patient populations is shown in Table 3 and further details regarding the selection process are displayed in the supplement S.3.

**Table 3:** List of pre-selected patient populations for series of systematic reviews

#	Health conditions	ICD-10 categories	Studies
1	Acute diarrhoea	A09.0	7
2	Insomnia	F51	10
3	Hypertension	I10	9
4	Female hormone dysregulation	N94.3, N94.4, N95, N95.3	16
5	Infections (respiratory tract infections, otitis media)	H66.9, J02.9, J06.9, J22	33
6	Allergic rhinitis	J30.1, J30.4	22
7	Musculoskeletal pain	M06.9, M16.9, M17.9, M19.9, M47.8, M54, M79.7	34
8	Asthma	J45.0, J45.9	15
9	Wound healing/pain	T14.9, Z48.8, Z98.8	39
10	Chronic sinusitis	J32.9	10
11	Influenza	J11.1	34
12	Diabetes mellitus type 2	E11.9	7
13	Complications during labour	O75.9	7

The patient populations were either defined by ICD-10 categories, with related classes being combined (e.g. J45.0 “predominantly allergic bronchial asthma” and J45.8 “mixed forms of bronchial asthma”) or determined from a homeopathic perspective as related (#4, #5, #7, and #9 in Table 3). Based on the preselection of patient populations in Table 3, SRs will be conducted over the course of the project, with the topics and the number of SRs determined by the published results of other research groups and the available resources

### Objectives

The objectives are (a) to assess the efficacy, effectiveness, and safety of homeopathy for prevention and/or therapy of diseases in patient populations selected on the basis of a health condition, (b) to assess internal, model, external validities, and quality of reporting, (c) to investigate components of the intervention complexity, and (d) to identify research gaps.

Depending on the specific health condition, the SRs will address hypotheses about single outcome comparisons and those reflecting a holistic perspective. Below, we provide possible examples for specific hypotheses.

o The mean efficacy of HPs is greater than that of placebo for reducing the symptoms of a given disease.
o The mean effectiveness of HPs is equal to that of active controls for reducing the symptoms of a given disease.
o The probability of harms in patients treated with HPs is smaller than in patients treated with an active control.

Possibilities for general hypothesis:

o Contextual factors of HP interventions (e.g. individualization vs. non-individualization) impact the size of the outcome effect estimates.
o Patient satisfaction is higher for patients treated with HP compared to the control.
o The study design and/or the internal, external, model validities of the studies are associated with the size of the effect estimates.

## Methods

The protocol is written in accordance with PRISMA-P [49]. We will follow the Cochrane Handbook of Systematic Reviews, the Methodological Expectations of Cochrane Intervention Reviews (MECIR) [46, 50] and the Sum-HomIS recommendations which provide a consensus on how systematic reviews and meta-analyses of HPs should be conducted in view of a high level of heterogeneity of the included studies [48].

The reporting of SRs will follow PRISMA 2020 [51] and its extensions PRISMA for complex interventions [52] and PRISMA harms [53]. The patient involvement will be reported using the GRIPP2 checklist [54].

In addition, the results are to be presented in such a way that they meet the requirements of AMSTAR-2 [55] and ROBIS [56] and have an objectively neutral presentation, in particular avoiding spins (i.e. a description that overstates/understates efficacy and/or harm) [57].

This protocol template will be used to derive topic-specific protocols which will be registered on the international prospective register of systematic reviews (PROSPERO, www.crd.york.ac.uk/prospero/).

### Review teams

For each of the SRs, a team consisting of at least two methodologists and at least two clinicians will be formed. Of the clinicians, one should have practical experience in homeopathy, and the other should bring expertise regarding the patient populations to be studied. The methodologists must have experience in conducting SRs and MAs, including the use of study quality assessment tools and the evaluation of the certainty of evidence. Alongside the core team, a scientific advisory board consisting of individuals with methodological expertise in SRs, clinical practice experience, and knowledge of homeopathy will support and monitor the SRs. We will integrate topic-specific experts into the process (e.g. statisticians for Bayesian MA) to ensure methodological quality and contextual relevance.

### Criteria for considering studies

#### Types of participants and setting

People of any age will be eligible if they are at risk of a disease or suffer from a disease or condition and have the opportunity to be treated with a HP in one of the preselected topic areas (see Table 3). We will include any setting (e.g. primary care, hospital, self-medication).

#### Types of interventions

We will include studies if one of the groups receives any type of homeopathic treatment involving the delivery of a HP with all routes of administration accepted, such as oral application, inhalation, injection, or application through the skin, eye, or ear. The intervention can be individualized homeopathy selected by a homeopath following a consultation or non‐individualized HP delivered without a consultation. This broad definition reflects the diversity of homeopathic interventions in clinical research; potential heterogeneity will be explored in subgroup analyses (Investigation of heterogeneity section). HP can include both single substances or complex preparations with more than one substance. HPs are eligible for inclusion if they are prepared in accordance with a nationally recognized homeopathic pharmacopoeia and are used according to homeopathic principles, as evidenced by application based on a homeopathic materia medica or repertory. Products prepared homeopathically (e.g. mother tinctures) but used outside the homeopathic context—such as for phytotherapeutic or conventional pharmacological indications—will be excluded. The HP can be delivered either alone or as add-on to one or more than one other active treatment(s), but only if a control group is treated with the exact set of treatments without homeopathy, i.e. if it is possible to draw conclusions about the relative effectiveness of the HP tested. We will include all remedies if they fulfill the criteria of a homeopathic medicinal product. Interventions which do not match the definition of an HP will be excluded. Studies comparing combinations of HPs and another intervention with a third therapy will also be excluded.

#### Types of comparators

All controls will be included unless two or more HPs are compared with each other, but without a non-homeopathic control. The controls may include placebo, no intervention, waiting list, and active interventions (usual care or others).

#### Types of outcome measures

Outcomes of interest are related to clinical efficacy and effectiveness and safety. Since core outcome sets (COS), defined as a consensus-based minimum set of outcomes that should be measured and reported in all trials for a specific health condition [58], are rare in homeopathic research, the outcomes and the ranking of their relative importance will be defined for each SR individually by the review team a priori.

In order to examine the complexity of the intervention, we will extract up to seven critical and important outcomes per study [59]. All outcome parameters that reflect the efficacy, the effectiveness, or the safety of the HP in relation to a control are eligible for inclusion. These can be objective and subjective measures. They may cover mortality, morbidity, symptom relief or disease severity, duration of illness, quality of life and other patient-reported outcome measures (PROMs), adverse events, and surrogate parameters (e.g. body mass index for weight loss or gain) and related outcomes such as consumption of medication. For each of the condition-specific SRs, the outcomes to be analyzed are determined according to the following procedure:

Step 1: During the protocol stage, the research questions and primary outcomes are developed a priori by the review team. This process is guided by the template protocol, the clinical and methodological expertise of the team, the corresponding Cochrane Handbook chapter 9 [60], and published SRs. To ensure that relevant outcome parameters are transparently prioritized:

- A comparison will be made with current SRs, preferably Cochrane reviews, on the indication in question.
- We will search for COS in the COMET database (comet-initiative.org) [58].
- We will focus on patient-centered outcomes (ichom.org).
- The relative importance of outcomes is assessed based on the GRADE recommendation [61] and the nature of the intervention.
- We will document the outcome selection within the indication-specific protocol.

Step 2a: A comprehensive literature search is conducted, followed by the selection of studies and a preliminary data extraction of study characteristics. All available outcomes per study will be extracted and presented as “characteristics of included studies”. The review team assesses the usefulness of conducting a MA for addressing the overall questions. This decision is based on the statistical combinability of the studies and substantive issues. These refer to the research questions of the respective review and may include patient groups, interventions, outcomes, and results (see Lopez [62], Table 1 and [59]).

To investigate the systemic nature of homeopathy, we plan to pool multiple outcomes as “overall effectiveness” in exploratory analyses if the studies are homogeneous enough for pooling. Outcomes will qualify for inclusion in “overall effectiveness” if they measure any type of symptom change such as symptom severity, duration of symptoms, duration of disease, or response rate.

Step 2b: The review team specifies the grouping, outcomes, and comparisons based on the Intervention Synthesis Questions (InSynQ) guideline [63]. If any additions or changes are needed compared to the initial plan in step 1, these adjustments are documented.

We will exclude studies if the outcomes of interest were neither explicit measures of change nor measured at least twice, i.e. at baseline or before the intervention and at least one time after the beginning of the intervention. We will also exclude studies if they did not measure an outcome of interest (e.g. only measured the costs of an HP intervention).

#### Types of studies

In order to better reflect the long-term outcomes, and the safety and the complexity of homeopathy in a real-world setting, we will include prospective, non‐randomized controlled studies of interventions (NRSIs) in addition to randomized trials (RCTs) and cluster‐randomized trials (cRCTs). This follows methodological articles and guidelines acknowledging the relevance of NRSIs in SRs [64, 65]. We will apply the following algorithm to decide whether to include NRSIs or not (Fig. 24.1.a, [65]): if there are insufficient RCTs addressing the PICO or if the RCTs address the PICO indirectly AND if the available NRSI have the study design features to address the PICO without critical risk of bias AND if the available NRSIs address the PICO directly, the NRSI will be included in the review.

We will also include n-of-1 trials (in which a single participant receives both HP and the control intervention in a randomly allocated sequence) if randomization and blinding are performed [66].

We will exclude cross-sectional studies (without two or more points in time when outcomes are measured), retrospective cohort studies, case–control studies, before-after-studies (evaluation of a single group of participants at two time points), interrupted time series (as a special case of before-after-studies), case series, and case reports (see [67] for an overview of NRSIs), animal studies and protocols. We will also exclude publications which were retracted.

Data from published (full‐text articles and conference abstracts) and unpublished (grey literature) eligible studies will be included. No studies will be excluded for language reasons. Studies in languages other than English and German will be translated using DeepL.com, and the translations will be compared. In case of doubt, native speakers will be consulted for a translation.

### Search methods for identification of studies

The aim of the SRs is to create a picture as comprehensive as possible of the current data situation on the relative effectiveness of homeopathic interventions for the treatment and prevention of health conditions.

The literature search will follow chapter 4 of the Cochrane Handbook for Systematic Reviews of Interventions [68] and its technical supplement [69]. To ensure that all available literature is identified, we will implement a two-way approach by (A) building on the HOMIS database, a living bibliography of comparative homeopathic intervention studies [36], and (B) conducting a separate, peer-reviewed literature search for each of the conditions in question.

As a consequence, the search strategy has multiple redundancies. The overall search strategy was developed by one reviewer and one librarian and information specialist independently, including a peer-review process based on the PRESS guideline [70]. The search of the topic-specific SRs will be conducted independently by two reviewers. If no consensus can be reached through discussion between the two reviewers, a third reviewer will mediate. This process is carried out in parallel with the similar approach being used to maintain the HOMIS database. The review team will include content experts in the field who will be provide potentially relevant additional literature.

#### Electronic databases

The primary source for searching for suitable studies is the bibliography of homeopathic intervention studies in human diseases (HOMIS) [36]. This maps the status quo of clinical research in homeopathy, is based on a continuously updated, comprehensive literature search, and covers both publications via academic journals and grey literature by a multitude of sources (see [36] for details).

In addition, the following databases will be screened from their respective inception dates up to the day of the search.

General:

- MEDLINE (via Ovid)
- EMBASE (via Ovid)
- CINAHL (via EBSCOHost) – Cumulative Index to Nursing and Allied Health Literature
- Cochrane Central Register of Controlled Trials (via Cochrane Library)

Complementary medicine:

- AMED (via Ovid) – coverage of Allied and Complementary Medicine
- TMGO (via Bireme) – WHO Traditional Medicine Global Library (beta version available at https://staging.tmgl.org)

Regional:

- AYUSH Research Portal—India
- LILACS (via Bireme)—Latin America and the Caribbean countries

After consulting experts of Chinese medical literature, we decided to exclude Chinese databases due to lack of relevance.^2^

Topic-specific:

-  PsycINFO (via Ovid)—coverage of behavioural science and mental health.

#### Other sources

Other sources include journals whose publications may not be found via MEDLINE, reference lists, grey literature such as dissertations, conference papers, and preprint publications as well as search engines.

#### Citation index

The two main subscription citation indexes are Web of Science; OpenAlex and Google Scholar are two free additional information sources with a wide coverage of healthcare journal publications. Following the recommendation of the technical supplement of the chapter 4 of the Cochrane Handbook, however, a forward citation search is not a requirement for the search strategy [69]. We will therefore limit our search to Web of Science and a backward search.

#### Trial registers

To identify additional randomized trials [71] and additional information [72], registries are an important source. The following registers will be searched:

- US National Institutes of Health Ongoing Trials Register (clinicaltrials.gov).
- World Health Organization International Clinical Trials Registry platform (trialsearch.who.int).
- The EU Clinical Trials Registry (clinicaltrialsregister.eu).
- Clinical trials registry India (ctri.nic.in/Clinicaltrials/login.php).

### Other electronic sources

Additional resources will be incorporated in our search depending on preliminary searches and experts’ discussions. They include ProQuest Dissertations & Theses Citation Index, oatd.org, dart-europe.org for theses and dissertations and medRxiv.org and bioRxiv.org for preprint server.

#### Search strategy

A general search strategy (see supplement S.2.1) will be adapted to the respective information sources and indication-specific topics of the SRs. The search strings will be combined with the Boolean operators “AND” and “OR”. The search strategy will be developed for MEDLINE and translated into other database syntaxes with the help of the software *polyglot* (https://tera-tools.com/polyglot) [73]; the resulting search strings will be checked and amended, if necessary, to the database-specific search terms by hand.

1. The HOMIS database will be searched by ICD 10 code, by free text with MESH terms of the indication in question, and by screening the titles.
2. For Medline, Embase, CINAHL, the Cochrane Library, AMED, and further databases such as PsycINFO, search strategies will be developed according to the guidelines in chapter 4 of the Cochrane Handbook of Systematic Reviews [68, 69] and peer-reviewed as recommended by the PRESS statement [70]. The search strategy is informed by condition of interest and the intervention. To limit the search results, we may additionally use terms regarding the prospective, comparative types of studies.

### Homeopathy

For the development of the search strategy regarding the topic of homeopathy, we followed the strategies used for the HOMIS database [36] and for Cochrane systematic reviews on homeopathic interventions [32, 74, 75].

The initial searches included the terms “homeopathy” and alternative spellings “homeopath*”, “homoeopath*”, “homoop*”, “omeop*”, and “homopath*”, “formularies”, “pharmacopoeias”, “materia medica”, “nosode”, “potentis*”, “potentiz*”, and terms related to complementary and alternative medicine such as “alternative medicine”, “complementary Therapies”, or “holistic health”.

### Randomized controlled trials

The Cochrane “highly sensitive search strategies for identifying randomized trials” will be used for Medline and EMBASE to identify RCTs [69].

### Non-randomized studies of interventions

Searching for NRSIs has been reported to be less straightforward than searching for randomized trials [65]. Following the chapter 24 of the Cochrane Handbook of Systematic Reviews, we will therefore combine search terms that are used as filters for validated, high sensitivity searches for NRSIs [76] and “snowballing” methods [65].

### Patient populations

The search strategies for conditions will be developed according to [68, 69] and, if available, based on Cochrane systematic reviews (e.g. [77, 78]).

3. The LILACS search strategy was developed following one of the few (according to [79]) Cochrane Reviews reporting respective details regarding the regional database [80].
4. The AYUSH Research Portal and the Web of Science will be searched by combining search terms for the condition in question and homeopathy.

Details regarding the databases, the corresponding search interfaces, and examples of the database-dependant search strategy are displayed in the supplement (see suppl. S.2.2).

We will not conduct a separate search for studies on harms and will only include those harms that are reported in the identified studies.

The searches will span the duration from the earliest possible date, depending on the database, to the day of the literature search.

The search strategy concept and database-specific demonstration searches are displayed in the supplement (see suppl. S.2.1).

#### Documentation of the search

The literature searches will be documented according to PRISMA-S [81]. The PRISMA-S checklist will be made available as a supplement of the published review. The *PRISMA2020* R package (or the corresponding Shiny app) will be used to design PRISMA-compliant flowcharts [82].

### Study selection

Data records of the references obtained by the literature search will be managed with Covidence systematic reviews software (Veritas Health Innovation, Melbourne, Australia; www.covidence.org). After removal of duplicates and a manual check, the titles will be screened by two reviewers independently, and reports will be sought for retrieval and assessed for eligibility by two reviewers. The study selection is carried out in Covidence. For studies excluded after full text screening, we will document the reference and justify the reason for each study’s exclusion. If no consensus can be reached through discussion between the two reviewers, a third reviewer will mediate.

### Data collection and management

#### Data collection process

The data extraction process will be a two-stage process. In a first step, the following information will be collected: study ID, population, homeopathic intervention type, HPs, control, ICD-10 category, and outcome parameters. These are used by the review team to define PICO questions and prioritize outcome parameters.

During the second stage, two reviewers will independently extract study characteristics and outcome parameters using Covidence and Microsoft Excel (Microsoft Corporation, Redmond, USA). The extracted data will be automatically assessed for differences by Covidence. Inter-extractor reliability will be calculated for the primary outcome parameter. Extraction tables and coding aids will be developed in advance (see supplementary files), adapted for the single SRs and piloted with up to three studies each beforehand to reveal ambiguities, missing values and other shortcomings.

The extractions will be reviewed and compared with each other. Differences will be resolved through discussion. If no consensus can be reached through discussion between the two reviewers, a third reviewer will mediate.

#### Data extraction

The codebooks of the SRs will encompass three dimensions, which include (A) characteristics of the included studies, (B) intervention details, (C) factors of complexity, as well as (D) estimated outcome effects. They will be developed in accordance with the quality criteria of AMSTAR-2, ROBIS, the PRISMA guidelines, and examples from other condition-specific SRs (see supplementary files for preliminary drafts).

For included studies, we will extract information regarding:

A. Characteristics of the included study:

- The publication (citation, country, year of study conduct).
- Participant characteristics (age, sex, severity or stage of illness, comorbidities, co-medications, treatment preferences).
- Study characteristics (study design, duration, sample size per arm, numbers randomized per arm, withdrawals, intention to treat (ITT) analysis, setting, funding sources, missing data).

B. Details of the intervention:

- Type of homeopathic intervention (classical/individualized, isopathy, clinical, isopathy, complex).
- Ingredients of all HPs applied (all substances with potency levels).
- Manufacturer.
- Frequency.
- Existence and type of co-interventions.
- Purpose (preventive, therapeutic).
- Frequency and any changes of the co-intervention.
- Control (e.g. active control (including dose, duration), placebo, no-treatment control, add-on, waiting-list).
- Measures taken against cross-contamination between HP and control.

C. a modified version of the Intervention Complexity Assessment Tool for Systematic Reviews (iCAT_SR) [83] that covers homeopathy-relevant dimensions including the degree of individualization (see chapter 6.8).

D. effect estimates: outcome (e.g. health status), measurement instrument (e.g. WOMAC), type of outcome (e.g. mean change from baseline), time point of measurement, absolute change for each arm (n/N (%) or mean (SD)), duration of intervention.

In anticipation of diverse study data, we will select summary statistics according to a pre-determined order of preference, reported by Daly et al. (2021a, b) for continuous outcomes [84]. For example, the mean difference (MD) and its standard error (SE(MD)) from ANCOVA will be preferred over change-from-baseline means (CFB) per arm and its standard deviation (SD(CFB)), and CFB over MD and SE(MD) at follow-up (see Table 4.1 in [84] for details).

For event data, we will use log hazard ratios if event rates change over time [85]. Otherwise, log odds ratios will be preferred to log relative risks unless there is evidence that the latter are less heterogeneous [85].

For ordinal data, the choice of analysis depends on the reporting of the studies; therefore, all forms of ordinal effect sizes are to be extracted to decide after extraction whether dichotomization will be performed or analysis as a continuous value will be conducted [86].

#### Dealing with missing data

If data are missing or if inconsistencies in the reported data of the underlying studies are identified during data extraction (e.g. multiple conflicting effect sizes), the study authors will be contacted to request a reliable effect size. In detail, the corresponding authors of the respective studies will be contacted via email. If there is no response, a reminder is sent after 10 days. If this also remains unanswered for 10 days, the value will be documented as missing. Data from retracted studies will not be used.

#### Imputation of missing values

Missing standard deviations for the change from baseline will be imputed according to the Cochrane Handbook chapter 6.5.2.8 [86]. The mean correlation coefficient for this imputation will be estimated with Corr = 0.5 according to Fu et al. [87].

Missing means and their corresponding standard deviations will be estimated from the sample size, median, range and/or interquartile range according to Wan et al. [88] and their supplementary Excel spread sheet including all formulas [88]. If only *p*-values or *t*-values are available, missing standard deviations will be calculated with Review Manager 5.4 [89].

Studies with missing data will be included in the systematic review and evaluated narratively if imputation is not possible. If missing data are imputed, the impact of the imputation will be examined in sensitivity analyses, comparing it to a “complete case analysis” [90].

If further imputations or study-specific decisions need to be made during the data extraction that could not be planned in advance, the decision will be documented and made publicly available as part of the final report to ensure transparency and reproducibility.

### Risk of bias assessment

The risk of bias will be assessed by the team of reviewers (see “review team”). ROB-2 and ROBINS-I will each be implemented by two reviewers, with both having experience in assessing the instruments. MVHT and RITES will also be carried out by two reviewers, with at least one being a clinician with experience in homeopathy. Risk of bias and model validity represent complementary methodological dimensions and will therefore be assessed separately and considered jointly during the interpretation of results.

#### RoB-2

To assess the risk of bias of the included RCTs, two reviewers will independently use the Cochrane Collaboration Risk of Bias Tool (RoB2) [91]. In this way, we can investigate possible biases in the randomization process, due to deviations from the intended intervention, due to missing data outcome, in the measurement of the outcome, and in the selection of the reported outcomes. The domain-specific and overall assessments will be categorized as low, of some concern, or high risk of bias.

If multiple outcomes are included in the meta-analysis, RoB2 will be conducted separately for each outcome. If multiple outcomes are pooled, resulting in one effect estimate, the corresponding ROB2 will represent a conservative estimate of the risk of bias; conflicting evidence will be documented.

As part of the assessment of selective reporting of outcomes, we will check whether only significant results were published and whether non-significant results were summarized generically (e.g. “*p* > 0.05”).

For cluster-randomized trials and crossover trials, the respective adaptations of the RoB2 tool and detailed guidance will be applied [92].

#### ROBINS-I V2

NRSIs will be assessed independently by two reviewers using the Cochrane ROBINS-I V2 tool [93, 94].

We will assess the risks of bias in seven domains due to confounding, in classification of interventions, in selection of participants into the study (or into the analysis), due to deviations from intended interventions, due to missing data, arising from measurement of the outcome, and in selection of the reported result. The domain-specific and overall assessments will be categorized as low, moderate, serious, or critical. For multiple outcomes, ROBINS-I will be conducted as described for ROB2.

For both ROB2 and ROBINS-I, the assessments will be conducted outcome-specific. To reduce inconsistency in judgments, we will pilot the tools with two reviewers and develop topic-specific support documents which amend the published guidelines [95, 96]; if necessary, this procedure will be repeated to yield a topic-specific document.

### Model validity and external validity

To supplement RoB-2 and ROBINS-I, the included studies will also be evaluated with established instruments covering methodological quality and homeopathy-specific aspects. Model validity of the homeopathic intervention will be evaluated with the Model Validity of Homeopathic Treatment (MVHT) instrument [97]. The efficacy-effectiveness spectrum will be captured with the Rating of Included Trials on the Efficacy–Effectiveness Spectrum RITES instrument [98]. For NRSIs, MVHT and RITES will also be applied in an exploratory manner, recognizing that neither has been formally validated for non-randomized designs [99]. Both instruments will be independently assessed by two raters with clinical experience; any discrepancies will be resolved through discussion, with a third reviewer consulted if needed.

For the assessment of MVHT, raters will select the outcome that is reported in the respective study and holds the highest priority within the indication-specific SR protocol’s outcome hierarchy with the exclusion of safety. If multiple outcomes of the same hierarchy level are reported, raters will select the first outcome listed in the protocol. Raters must agree on the selected outcome, which will be documented in a standardized extraction template. Raters will indicate whether the selected outcome differs from the study’s primary outcome (yes/no). If yes, the primary outcome of the study will be documented. Frequent discrepancies will be reported and considered in the interpretation of MVHT results.

### Assessing complexity

In protocol version V1, we had planned to apply an iCAT_SR framework with five dimensions across two systematic reviews. A pilot application, however, showed strong redundancy among several domains: provider skill, inter-component interaction, and dependence on individual-level factors were highly correlated with the degree of individualization. To increase feasibility and informativeness, we therefore refined the approach.

In the current protocol, we restrict the assessment to two iCAT_SR dimensions—number of active components and degree of tailoring/individualization—which capture the main axes of variation while avoiding overlap. Both domains will be extracted independently by two reviewers with consensus adjudication. Details of operationalization are provided in the Supplement (S.4).

### Measures of treatment effect

The effect measure will be selected in accordance with the Cochrane Handbook of Systematic Reviews chapter 10.4 for dichotomous data and chapter 10.5 for continuous data [90].

For dichotomous data and time-to-event data, we will apply odds ratios (OR) and hazard ratios (HR), respectively. Count data will be presented as a rate ratio. All effect estimates will be reported with 95% confidence intervals (CIs). Continuous data will be reported as standardized mean difference (SMD) using Hedges’ g formula along with a standard deviation (SD) where different scales are used to measure the outcome. Otherwise, mean differences with SDs will be applied. To ensure that all measurement scales show the relative effects in a common direction, the direction of effect estimates will be aligned across studies by multiplying by (− 1) if necessary. For continuous data, a comparative treatment benefit of HP over the control will be indicated by an SMD > 0. MD will be used according to the natural sign of each scale, preserving the inherent interpretability of the instrument. For dichotomous data, a relative advantage of HP compared to the control will be demonstrated by an OR < 1.

If continuous data are used in the MA and studies present results as dichotomous data, these may be converted to SMD and vice versa [100, 101]. If data are reported in a format that is not suitable for inclusion in a MA, they will be converted accordingly [86].

Since intention-to-treat (ITT) analysis represent a more pragmatic and clinically relevant approach compared to the per-protocol (PP) design [102], we will preferentially extract results based on ITT analyses where these are reported by the study authors.

If NRSIs report adjusted or unadjusted effect estimates, we will use adjusted values [65]. If multiple adjusted estimates are reported, we will choose the one that minimizes the risk of bias of confounding based on discussion in the review team. This may be the effect size based on the model named by the authors as primary or based on a different model. The latter case would be documented with reasons as well as the variables used for the adjustment.

Since our aim is to investigate the existence of a difference between the clinical effectiveness of the HP and that of controls, we define a null-threshold and will not specify indication-dependent effect magnitudes for interpretation; thus, we determine the certainty of evidence in a non-contextualized manner.

### Unit-of-analysis issues

We expect most studies to randomize patients at the individual level, meaning that each participant will receive one intervention compared to another control intervention. In the case of the following study designs, we will follow chapter 23 of the Cochrane Handbook [103]:

o If a study includes more than two relevant arms, the arms will be combined to enable pairwise comparisons. Alternatively, a control group will be split if it is shared by two intervention groups.
o Cluster-randomized studies, in which randomization involves groups of participants, will only be included if the analysis is adjusted for the cluster design.
o If we identify studies with a cross-over design, where participants are assigned to a sequence of different interventions, only the first phase will be included to avoid carry-over effects of the intervention. Later phases will be investigated in sensitivity analyses.

For cluster-randomized trials and cross-over studies, the risk of bias assessment will be based on the respective specialized ROB-2 tools.

In case of repeated measurements, we will choose one of two strategies depending on the included studies and available information from other studies. We will either define different outcomes by categorizing time frames (e.g. short-term, medium-term, long-term) or select the longest time point unless the clinical relevance indicates another time point.

To address other cases of data multiplicity, we will follow the three steps approach suggested by López-López et al. [62].

### Assessment of heterogeneity

Heterogeneity, or variability between studies, is caused by clinical and methodological diversity. Both are assumed to be high due to the variability in populations, settings, interventions, controls, and outcomes. Because, if left unexplained, it limits the validity of the MA, we will address and measure it in accordance with recommendations [90, 104, 105]: Each SR will be conducted by a review team with different areas of expertise and will decide to undertake MA for the primary analysis of clinical efficacy or effectiveness only if the PICO elements and other factors are judged to be sufficiently similar to ensure an answer that is clinically meaningful [90]. This assessment will be based on prior topic specific SR, clinical expertise and guidelines [106]. Studies with substantial methodological differences will be analyzed by other data synthesis methods, i.e. SWiM [107] and pooled in exploratory investigations only.

To evaluate the degree of heterogeneity, we will first compare the studies based on the aforementioned factors. Subsequently, we will visually examine forest plots regarding the overlap of Cis and will assess heterogeneity among the studies using the chi^2^ test and quantify it through the heterogeneity index (*I*^2^), the heterogeneity variance (*τ*^2^), and a prediction interval [108, 109]. *I*^2^ values will be categorized as follows: 0%–40% may represent little heterogeneity, 30%–60% may represent moderate heterogeneity, 50%–90% may represent substantial heterogeneity and 75%–100% represent considerable heterogeneity [90]. We will use a cut-off value of *p* < 0.10 to decide on the statistical significance of the chi^2^ test. Categorization of heterogeneity will not be applied universally based on *I*^2^ [90] but interpreted in the clinical context and with regard to the direction and magnitude of the effect estimate [110]. We plan to explore potential sources of heterogeneity by reconsidering the effect measure, excluding outliers, and subgroup analyses and meta-regression (see data synthesis) [90].

### Data synthesis

The strategy of the data synthesis follows the objective to investigate homeopathy as a whole medical system. We will follow both a broad and a narrow analytical focus. Thus, we aim to conduct pairwise comparisons between HP and controls to assess relative efficacy, effectiveness, and harms. In addition, we plan to explore the complexity of the intervention and identify patterns in the heterogeneous studies, with the goal of drawing conclusions about which factors influence the effect sizes under investigation or determining what is missing to enable such conclusions. If MA is feasible, we will use a frequentist approach to guarantee model simplicity, conventional statistical norms, and familiar, well-established interpretations. In order to make use of prior knowledge and model flexibility, Bayesian meta-analysis (BMA) will also be used in addition, depending on the data.

Each MA will be planned by a distinct review team. If the studies and data are sufficiently complete and methodologically and clinically homogeneous, we will conduct a MA. The MAs will be conducted independently by two reviewers. The analyses regarding subgroups, robustness against outliers, heterogeneity, and publication bias will be calculated only once and checked by the review team. The BMA will be checked by a statistician with experience in performing BMA.

We will analyze outcome data using a pairwise random-effects model due to the expected heterogeneity of studies. We will employ a fixed-effects model only in cases when all studies are assumed to represent patients from the same population or when the analysis aims to draw inferences strictly limited to the included studies without generalizing beyond them [111]. Patient populations, homeopathy interventions, controls, and outcomes will be combined based on clinical guidance, multiple assessments by different reviewers and discussion. All analyses will be stratified regarding the study design, i.e. RCTs and NRSIs will be pooled separately [90]. Outcomes from NRSIs that are determined to have an overall critical risk of bias, as evaluated using ROBINS-I, will be excluded from the MA.

The following comparisons are examples of analyses which may be specified for the single SRs with regard to timing, setting, patients, and outcomes:

o HP versus placebo.
o HP versus active controls.
o HP versus no treatment.

For continuous outcomes, we will use the restricted maximum likelihood estimator and the Paule-Mandel estimator when the number of studies is small, to calculate the heterogeneity variance *τ*2 [109].

In the case of dichotomous data, we will apply the Paule-Mandel estimator for the between-study variance for random-effects models if there are no zero cells [85]. If there are zero cells, Mantel–Haenszel estimates will be used for fixed-effects models and Bayesian methods for random-effects models.

The confidence interval around the pooled effect estimate will be calculated with the Knapp-Hartung adjustment [112]. In the case of few studies (*N* ≤ 5) with unequal sizes, different estimators will be tested to validate the robustness of the analyses. Studies using individualized homeopathic interventions will be visually indicated in forest plots to support the interpretation of subgroup differences.

Depending on the data, a multi-level MA will be used to account for possible dependencies among effect estimates from the same study. Sampling variance of individual effects (level 1), and variance of effects within (level 2) and between (level 3) studies will be incorporated into the model [109, 113–116]. The variance–covariance matrix will be approximated based on an assumed intra-study correlation coefficient and alternative assumptions will be tested to guard against model-misspecification, e.g. the robust variance estimation [117].

The data synthesis will be carried out by one analyst, while an independent analyst who is not part of the review team will calculate the meta-analyses in parts (e.g. main outcomes defined in the specific protocol). The results will be compared, and divergent synthesis decisions will be resolved through discussion or mediation by a third reviewer. Since this project is a series of reviews and the template protocol will be published in advance, it cannot be ruled out that the external analyst might be aware of the hypotheses. Thus, blinding of the synthesis process cannot be ensured.

#### Sensitivity analysis

We plan to conduct sensitivity analyses to examine the robustness of the MAs and will exclude, for example, the following studies to determine whether their exclusion alters the findings:

- Studies at high or serious risk of bias as assessed with RoB2 and ROBINS-I, respectively.
- Outlier studies in the case of substantial statistical heterogeneity (*I*^2^ > 75%).
- Studies that differ from the others in clinical or methodological aspects (e.g. self-medication).
- Studies with missing data.

To validate the data extraction, forest plots will be visually inspected for outliers. If outliers are identified, the data extraction is rechecked (e.g. standard error reported as standard deviation), followed by an influence analysis. Studies with extreme influence are to be excluded, and heterogeneity patterns examined using a Graphic Display of Heterogeneity (GOSH) plot [110].

Depending on the pool of included studies, further analyses may help to check how reliable our results are when repeated with different models and to explore the circumstances under which homeopathy works [118]. For this purpose, BMAs (Bayesian meta-analyses) provide a flexible framework for computing complex models and [119] shift the focus away from significance to dispersion in effect size (as demanded for MA [111]). There is evidence that BMAs are reliable when cautious interpretation is required with frequentist MA due to few studies and unbalanced study sizes [120], which is common for the HPs [36]. In case of small studies where heterogeneity is often more pronounced, BMAs excel in estimating between-study variance (*τ*^2^) and the meta-analytic effect (µ) [121]. BMAs directly indicate the probability that the relative effectiveness is greater than zero (or another value), offering an intuitive measure of the current evidence for various stakeholders, including clinicians and patients [122].

Limitations can be seen in the computational complexity, the risk of model misspecification, as well as the dominance and familiarity of frequentist approaches in the field of MA, which necessitates additional explanations, e.g. of the posterior distribution or credible intervals. The most common criticism of the Bayesian approach, however, is that the priors are chosen subjectively, which may introduce bias into the effect estimates [123]. While in theory, prior elicitation translates domain knowledge into priors, there is a significant gap in the development of effective elicitation methods and evaluation techniques [124].

Therefore, we will follow recommendations and use non-informative priors for the overall mean effect and weakly informative priors (e.g. Half-Cauchy) for the heterogeneity distribution [125]. Additional priors, including informative ones based on expert opinion or historical evidence, may be used for exploratory purposes.

To address the potential impact of priors as well as the possible misinterpretation and improper reporting of Bayesian results, we will be following the When-to-Worry-and-How-to-Avoid-the-Misuse-of-Bayesian-Statistics (WAMBS) checklist for Bayesian statistics [126].

More specific approaches may be used depending on the question and the data situation, for example, model-averaged BMA addressing publication bias [127] and bias-adjusted BMA to combine RCTs and NRSIs [128, 129]. The BMA will be reviewed by an external statistician with experience in BMA and who is unaware of the SR-specific hypotheses.

#### Systematic review without meta-analysis (SWiM)

If clinical or methodological differences argue against synthesizing the studies using a MA as the primary outcome of the systematic review, we may modify planned comparisons and provide rationale for post-hoc changes according to McKenzie and Brennan [130].

Alternatively, we will use a systematic review without meta-analysis (SWiM) [107] and narratively analyze the studies or apply another synthesis method [130], i.e. group studies, describe metrics, summarize effect estimates or combine *p*-values, if possible, prioritize results, explain the sources of heterogeneity, assess the certainty of evidence, and present the results with graphical or tabular methods [107].

### Investigation of heterogeneity

Given the diversity of study designs, settings, and intervention modalities in the homeopathy literature, substantial heterogeneity is anticipated and will be explored systematically using subgroup analyses and sensitivity analyses.

We will explore the potential sources of heterogeneity with predefined subgroup analyses provided that at least 10 studies are available, following the Instrument for assessing the Credibility of Effect Modification Analyses (ICEMAN) [131]. To avoid ecological bias, we will not use aggregate study information (e.g. mean age) to categorize subgroups [109].

Particular attention will be given to sources of clinical heterogeneity related to the characteristics of the homeopathic intervention. Prespecified subgroup analyses will therefore examine the degree of individualization (individualized vs. non-individualized interventions). In addition, exploratory analyses may consider the distinction between single and complex preparations. If meaningful differences between subgroups are observed, pooled estimates across all intervention types will be interpreted with caution and contextualized accordingly. Further effect moderators will be defined for specific indications and/or investigated exploratively. The rationale for considering these subgroups is that different intervention characteristics, timeframes, contextual factors, and comparators may alter the outcome effects and may therefore be relevant for health professionals and patients. Subgroup analyses regarding the risk of bias, model validity, different outcome and publication types may provide insight into potential sources of heterogeneity. As multiple subgroup comparisons may increase the risk of spurious findings, these analyses will be interpreted cautiously and primarily considered hypothesis-generating.

For meta-regression, we will also explore MAs in case of 10 or more studies and consider ordinal or continuous moderators such as the risk of bias, model validity, year of publication, homeopathic potencies, and timeframes.

### Assessment of reporting biases

We aim to minimize the risk of bias due to missing evidence by including results from other sources than published reports [132]. Nonetheless, a potential publication bias will be analyzed by the visual inspection of funnel plots [132]. In case 10 or more studies are included in a 2-level MA, tests for small study effects will be performed [133] using Egger’s regression intercept method for MD and SMD [134] and Peter’s test for binary outcome data [135]. If there are indications of a publication bias, we will apply further methods [109] such as the Duval and Tweedie’s trim and fill method [136] or PET-PEESE [137]. For multi-level meta-analyses, we will use an adapted version of Egger’s regression test, where the pooled effect is regressed on some function of the standard error of effect sizes [138].

### Updating evidence

When new studies are published, there is a risk that SRs are no longer up-to-date and incorrectly reflect the current state of clinical research [139]. Therefore, updates may be necessary, which can take place sporadically or in the form of living systematic reviews (LSRs) which continually incorporate new evidence as it becomes available. Because this requires long-term resources, it only justified for questions of high relevance for decision-makers and for topics where new studies are likely to influence the existing evidence base [139].

Thus, LSRs may not be relevant for all indications to be examined and a decision will be made independently for each SR regarding whether an LSR will be initiated, how often it will be updated, and when it may be concluded. For organizational reasons, the LSRs will not be implemented as part of the initial publication of the SRs in peer-reviewed journals. Instead, the LSRs will be maintained on a separate website [140]. The methods will be documented according to PRISMA for LSRs [141] and adhere to the guidelines of the Cochrane Handbook of Systematic Reviews [139, 142].

### Software

The meta-analytical models are calculated with R 4.3.3 (or a later version) [143]. For example, we will use *metafor* [144] for conducting MA and may use *brms* [145], *metaBMA* [146] or *bayesmeta* [147] for conducting BMA. These tools will be applied in combination with others such as *tidyverse* and *dplyr*. A complete list of software used for the individual project will be documented with the final report of the respective SR.

### Assessment of the certainty of evidence

To assess the certainty of evidence of the MA, two reviewers will independently apply the Grading of Recommendations, Assessment, Development and Evaluation (GRADE) [148], with disagreement resolved by discussion or mediation through a third reviewer. The quality of evidence will be assessed for each effect estimate separately and rated as high, moderate, low, or very low [149].

Although evidence exists that pooling RCT and cohort studies would reduce low and very low GRADE ratings [150], proper guidance is missing when and how to integrate the evidence from RCTs and NRSIs, i.e. how respective GRADE rating should be combined in order to draw conclusions about an outcome assessed by both study types [151, 152]. Therefore, we will analyze RCTs and NRSIs in parallel and assess them using GRADE for the time being. The certainty of evidence will be assessed separately for RCTs and NRSIs, while their implications will be considered jointly during the interpretation of results [153]. Since ROBINS-I is a rigorous instrument for measuring the risk of bias in NRSIs, we will follow the GRADE guideline 18 and will start the rating for both RCTs and NRSI as “high” [154]. This approach will be disclosed in the summary of finding (SoF) tables.

The definitions of the quality of evidence levels are as follows (Table 1, [149]):

o High: we are very confident that the true effect lies close to that of the estimate of the effect.
o Moderate: we are moderately confident in the effect estimate: the true effect is likely to be close to the estimate of effect, but there is a possibility that it is substantially different.
o Low: our confidence in the effect estimate is limited; the true effect may be substantially different from the estimate of the effect.
o Very low: we have very little confidence in the effect estimate; the true effect is likely to be substantially different from the estimate of effect.

The certainty of evidence may be downgraded by one or more levels with regard to the risk of bias, inconsistency of results, the indirectness of evidence, the imprecision of effect estimates, and/or a publication bias. It may be upgraded due to criteria such as plausible residual confounding [149].

We define the target threshold for GRADE certainty ratings as non-null, given the contextual and implementation variability of complex interventions [155]. Where credible, outcome-specific minimally important differences (MIDs) are available, we proceed as follows [156]: If the point estimate is smaller than the MID, we revise to “little or no effect”. If it exceeds the MID, falls within a range of multiple MIDs, or if no credible MID is available, we retain the non-null target.

### Involvement of consumers

This protocol was developed in consultation with patient representatives, clinical and methodological experts, and by consulting scientific literature on the patient perspective (e.g. [157, 158]).

The scientific advisory board includes two physicians: an internist and gastroenterologist, and a general practitioner with extensive experience regarding the needs, preferences, and abilities of patients with various illnesses. This is complemented by the indication-specific expertise of the respective review team on which the discussion of each SR will be based.

Therapists with practical experience in the implementation of homeopathic interventions are involved in the entire process of all SRs, particularly in the selection of outcomes, grouping of studies for MA and the assessment of studies with ROB2, ROBINS-I and model validity. To further ensure the use and usefulness of the SRs, we will involve two patient representatives who can provide the homeopathic and conventional perspectives, respectively.

Fabienne Gigandet is a druggist and homeopath, as well as co-president of the patient-oriented organization Homeopathy Switzerland, which seeks to promote a responsible approach to personal health. Her personal interactions with patients, along with her understanding of their needs, expectations, and experiences, have contributed to the development of research questions, the selection of patient-centered outcome parameters, and the planned dissemination of the results.

Dr. Janney Wale holds a PhD in pharmacology, working in industry and academia. Following personal health challenges that prompted a reassessment of her priorities, Dr. Wale became a member of the Consumer Network within the evidence-based organization, The Cochrane Collaboration. She has since served as a prominent health consumer advocate in Australia. Her own experiences with illness, her expertise in science, and her role as part of the Cochrane Consumer Network will continuously contribute to shaping the review processes in a patient-oriented way.

Two reviewers conducted unstructured online interviews with FG and JW. In the interviews, patient populations, objectives, outcome parameters, and dissemination strategies were discussed. The feedback was documented and incorporated into the outcome selection and analysis strategy. For the series of SRs, details of the patient involvement are described in the supplementary information S.7.

We will report the patient involvement according to GRIPP2 [54] and follow best practice recommendations [159]. This series of SRs will help inform patients and their healthcare providers about the current evidence on the relative efficacy and safety of HP, supporting the identification of the best possible individual therapies.

### Protocol deviations

Differences in the reported methods between SRs and their protocols are prevalent [160]. Protocol deviations can, for instance, include a change of selection criteria, outcome characteristics, deletion or addition of outcomes, modification of the analyses, or the assessments of the risk of bias or the certainty of evidence [161]. For this series of SRs, we will adopt changes and adjust the template protocol in the topic-specific protocols a priori, and accordingly, if methodological recommendations change during the course of the project.

In contrast, post hoc protocol deviations may introduce bias [162], and therefore, they will be documented and reported according to Cochrane Handbook section I.1.5 [163]. This amendment will be published as supplementary information to the manuscript; it will cover the reason and timing of the changes to create an audit trail and guarantee transparency [164].

If possible, the impact of the deviations will be explored in sensitivity analyses. In addition, any change of the protocol will be described in the manuscript along with a rationale following PRISMA item 24c [51].

### Use of Artificial intelligence

Large language models (LLMs) may radically change the landscape of evidence synthesis [165]. Numerous tools are available to improve the efficiency of SRs [166, 167], but standardized frameworks for the development, evaluation and use of those are still lacking [168, 169]. Despite their considerable potential, “concerns about the appropriateness, transparency and trustworthiness of AI do exist” [170]. The applications are considered a potential source of various risks that may affect the reliability and credibility of the resulting SRs [167, 171].

Thus, if we apply artificial intelligence (AI)-based instruments in the course of the project, they will only be used in addition to the established procedures or in accordance with the consensus guidelines to guarantee that standards for reliable evidence synthesis are not compromised. In particular, we will follow the recommendations for evidence synthesists provided by RAISE [165].

### Dissemination and data sharing

To guarantee transparency and reproducibility, we will share the search syntax, the data and the analysis scripts in formats that can be analyzed in open software (e.g. CSV, R) and make them available on osf.io. In order to make the results as widely available as possible, we will publish the SRs peer-reviewed journals that grant open-access update our analyses on a publicly available website and further promote our findings in science (e.g. conferences) and in plain language for the public and patients in particular. The planned completion of the SR will take place in stages and will begin on May 1, 2025.

## Discussion

In view of the widespread use of HP and the uncertainty regarding the clinical benefit for individual indications, this series of SRs intends to investigate the efficacy, effectiveness and safety of HPs for selected indications and explore possible context factors under which HPs work.

Our methodological approach aims to address the holistic context in which HPs can be applied (i.e. in classical homeopathy which, in clinical practice, is not given in standardized forms, but in individually adapted, complex forms of treatment which include a wide range of approaches). Thus, we intend to incorporate all available RCTs and NRSIs including grey literature to expand the evidence base, reduce potential bias (e.g. publication bias), and identify research gaps in the area of homeopathic care such as, for example, specific HPs or HP selection strategies that are widely used in practice but underrepresented in studies. In addition, studies could be identified as exemplary and the significance of new methods such as target trial emulation may be clarified. Despite the additional use of NRSIs, we will still utilize only a portion of the available evidence by excluding certain study types (e.g. retrospective studies, case series).

We consider it a strength of our methodology that, in addition to assessing internal validity using RoB-2 or ROBINS-I, we will also evaluate model validity and pragmatism using MVHT and RITES. However, since we also plan to determine the “overall effectiveness” using multiple outcome parameters on symptom reduction, as well as conduct specific pairwise comparisons, and assess the reliability of the evidence synthesis using GRADE, a wide range of evaluations and metrics will need to be interrelated for each indication. The interpretation by clinicians, who possess both indication-specific knowledge and expertise in homeopathy, and contextualization through a framework study, are intended to help draw conclusions from these findings. While we also take the patient perspective into account during the planning of the studies, it cannot be guaranteed how much this perspective will be incorporated into each individual SR on an indication-specific basis.

The clinical and methodological heterogeneity of the included studies, along with other reasons outlined (see chapter 5.3), complicates evidence synthesis and the derivation of corresponding conclusions such as the identification of specific contextual factors. However, these investigations are exploratory, as we are also attempting to identify research gaps, for example, to pinpoint studies and study designs that could help to overcome methodological limitations in the future. Furthermore, controlled studies investigating HPs report a wide range of outcome parameters. Documenting, analyzing, and evaluating these in relation to the respective patient population represents an initial step toward formulating core outcome sets, which could serve to standardize future studies and improve comparability.

Our plan to keep the SRs “living” by continuously incorporating new studies depends on the preliminary nature and further development of the evidence for each indication. However, this approach could be particularly important for a controversial topic like homeopathy, as it allows a comprehensive and up-to-date overview of the current evidence to be available for discussion.

Despite the absence of a generally accepted mechanism of action, the use of HPs is widespread. To provide the necessary foundation for scientific, medical, and societal debate, we aim to gather, review, and analyze the data on an indication-specific basis. Ultimately, we aim to contribute to informed treatment decisions for the benefit of patients.

## Supplementary Information

Supplementary Material 1. Supplementary information [172, 173].

## Data Availability

No datasets were generated or analyzed during the development of the protocol. All relevant data from this study will be made publicly available via the Open Science Framework (OSF) when the study is completed and published.

## Abbreviations

AE: Adverse events
BMA: Bayesian meta-analysis
CI: Confidence interval
COMET: Core Outcome Measures in Effectiveness Trials
COS: Core outcome sets
EV: External validity
GRADE: Grading of Recommendations Assessment, Development and Evaluation
HP: Homeopathic preparation
HR: Hazard ratio
MA: Meta-analysis
MD: Mean difference
MV: Model validity
MVHT: Model validity of homeopathic treatment
NRSI: Non-randomized study of an intervention
OR: Odds ratios
PICO: Population, Intervention, Comparator, Outcome
PRISMA: Preferred Reporting Items for Systematic Reviews and Meta-Analyses
RCT: Randomized controlled trial
RITES: Rating of Included Trials on the Efficacy-Effectiveness Spectrum
RoB: Risk of bias
RR: Risk ratio
SD: Standard deviation
SE: Standard error
SMD: Standardized mean difference
SR: Systematic review
SWiM: Systematic review without meta-analysis

## Footnotes

1 Hereinafter, the term homeopathic preparation (HP) refers to all substances prepared in accordance with the principles outlined in a nationally recognized homeopathic pharmacopoeia, including but not limited to the European Pharmacopoeia, United States Homeopathic Pharmacopoeia, or other comparable Pharmacopoeias. This definition encompasses the full spectrum of homeopathically prepared substances, regardless of their legal or regulatory status as, e.g. “homeopathic medicinal products” in Europe. The aim is to ensure scientific inclusivity by capturing both registered products and unregistered but pharmacopoeially prepared substances, thereby promoting conceptual clarity and consistency across diverse clinical and regulatory contexts.

2 Personal communication on 08.01.2025 with Jianping Liu, Centre for Evidence-Based Chinese Medicine, Beijing University of Chinese Medicine, Beijing, China.
