# Supplementary material for: Clinical research on homeopathic preparations: protocol template for a series of systematic reviews
Source: Syst Rev. 2026 Apr 7;15:136. doi: 10.1186/s13643-026-03168-z (PMC13081291; doi:10.1186/s13643-026-03168-z)
Supplement: Supplementary file 1 — Supplementary Material 1. Supplementary information [172, 173]. [file 13643_2026_3168_MOESM1_ESM.docx]

Supplementary information

S.1 Search results for protocols of systematic reviews

In order to harmonize our SR with the projects of other groups and to avoid redundancies, we will be looking for protocols that plan SR on homeopathy on a quarterly basis. Multiple sources (e.g. PROSPERO, Cochrane library, JBI) were searched for “homeopathy” (registration sites, data repositories, other sources) or “homeopathy” alone or combined with “protocol” and “systematic review” (journals) on October 31st 2024 (172, 173) (see table S.1)

We selected protocols dealing with systematic reviews on homeopathy that were published 2017 or later and reported as ongoing. Based on the results (see table S.2), the initial search will be repeated on a quarterly basis covering PROSPERO, the Cochrane Library, OSF and google scholar.

Table S.1: Information sources searched for protocols of systematic reviews on homeopathy

| Name | URL |
| --- | --- |
| Systematic Review registration sites | |
| PROSPERO | [https://www.crd.york.ac.uk/prospero/](sps:urlprefix::https) |
| Cochrane Library | [https://www.cochranelibrary.com/search](sps:urlprefix::https) |
| JBI | [https://jbi.global/systematic-review-register](sps:urlprefix::https) |
| Research Registry: Registry of Systematic Reviews/Meta-Analyses | [https://www.researchregistry.com/browse-the-registry](sps:urlprefix::https) |
| INPLASY | [https://inplasy.com/](sps:urlprefix::https) |
| Non-specific registration sites | |
| Center for Open Science | [https://www.cos.io/initiatives/prereg](sps:urlprefix::https) |
| Protocols.io | [https://www.protocols.io/](sps:urlprefix::https) |
| Journals | |
| BMJ open | [https://bmjopen.bmj.com/search](sps:urlprefix::https) |
| Biomed central | [https://www.biomedcentral.com/](sps:urlprefix::https) |
| JMIR Research Protocols | [https://support.jmir.org/hc/en-us](sps:urlprefix::https) |
| World journal of meta-analysis | [https://www.wjgnet.com/2308-3840/index.htm](sps:urlprefix::https) |
| Data repositories | |
| Zenodo | [https://zenodo.org](sps:urlprefix::https) |
| Open Science Framework | [https://osf.io/search](sps:urlprefix::https) |
| Other sources | |
| Google scholar | [https://scholar.google.com](sps:urlprefix::https) |

26 protocols were found in PROSPERO and one in OSF on 01.05.2025 which were labeled as ongoing and had “homeopathy” in the title. Five of these have been published. An additional protocol was found at osf.io. The results are shown in table S.2.

Table S.2: Registered protocols with “homeopathy” in the title and labeled as ongoing (01.05.2025)

| # | Identifier | Title |
| --- | --- | --- |
| 1 | CRD42024550063 | The Effect of Homeopathy Intervention in Menopausal Women on Menopause Symptom Severity and Quality of Life: Systematic Review and Meta-analysis of Randomized Controlled Trials |
| 2 | CRD42024545450 | Efficacy of Individualized Homeopathic Treatment: An Updated Systematic Review and Meta-Analysis of Randomized, Placebo-Controlled Trials Published 2014–2023 |
| 3 | CRD42024541687 | Efficacy of Homeopathy in the Treatment of Mental Health in Patients with Depression: A Systematic Review |
| 4 | CRD42024543160 | The Effect of Homeopathy Applied to Breast Cancer Patients on Quality of Life: Systematic Review and Meta-analysis |
| 5 | CRD42024541164 | Effectiveness of Ayurveda, Yoga, Unani, Siddha, and Homeopathy (AYUSH) Interventions in Randomized Trials for Enhancing Symptom Management and Quality of Life in Palliative Care: A Systematic Review and Meta-Analysis |
| 6 | CRD42024506053 | Meta-analysis of controlled clinical homeopathic trials from 1980 to 2022 |
| 7 | CRD42023445301 | Effect of Homeopathic intervention in Insomnia: A Systematic Review and Meta-analysis |
| 8 | CRD42023443278 | Prophylactic effect of various homeopathic preparations in the prevention of COVID-19: a systematic review and network meta-analysis |
| 9 | CRD42023434119 | Effect of Homeopathic Interventions in Sports Medicine: A Systematic Review and Meta-analysis |
| 10 | CRD42023434862 | Efficacy of Homeopathic Interventions in Primary Dysmenorrhea: A Systematic Review and Meta-Analysis |
| 11 | CRD42022346433 | Homeopathy for preventing and treating health conditions: a protocol for an evidence evaluation |
| 12 | CRD42022323364 | Individualized homeopathic intervention in real world conditions vs. conventional medicine: Protocol for a systematic review and meta-analysis of prospective cohort studies |
| 13 | CRD42022309334 | Efficacy of homeopathic treatment in monotherapy and/or association with pharmacotherapy in patients with mood disorders: a systematic review and meta-analysis |
| 14 | CRD42021259208 | Systematic review of usefulness of homeopathy for treatment of Polycystic Ovarian Syndrome |
| 15 | CRD42021225100 | Therapeutic effects of homeopathic treatment on premenstrual syndrome: a systematic review and meta-analysis |
| 16 | CRD42020209661 | Efficacy of homeopathic treatment: systematic review of meta-analyses of randomised placebo-controlled homeopathy trials for any indication |
| 17 | CRD42020199720 | Homeopathy for Upper Respiratory Tract Infections in Children: A Systematic Review |
| 18 | CRD42020194418 | The great compendium of homeopathy in cancer, an updated of scientific evidence in a systematic review |
| 19 | CRD42019130254 | The use of antibiotics under homeopathic treatment – a systematic review |
| 20 | CRD42024627224 | Homeopathic Interventions for Postoperative Pain Management: A Systematic review and Meta-Analysis |
| 21 | CRD42019123389 | Overview of systematic reviews of clinical studies of homeopathic Arnica montana, using ROBIS and AMSTAR 2 assessment tools |
| 22 | CRD42025649258 | Homeopathy for sinusitis: A systematic review and meta-analysis |
| 23 | CRD42025649244 | Homeopathy for tonsillitis: A systematic review and meta-analysis |
| 24 | [https://osf.io/5jbsk](sps:urlprefix::https) | A systematic review of Homoeopathic research on Gout and Hyperuricemia: Road ahead |
| 25 | CRD42025649244 | Homeopathy for tonsillitis: A systematic review and meta-analysis |
| 26 | CRD42025649258 | Homeopathy for sinusitis: A systematic review and meta-analysis |
| 27 | CRD420251036526 | Evaluation of Study Quality and Clinical Outcomes of Homeopathic Interventions for Warts: A Systematic Review |
|  | CRD42018096189 | Homeopathic remedies in the treatment of psychiatric disorders: a meta-analysis of randomized clinical trials [published 06/2020] |
|  | CRD42018087184 | Homeopathic remedies in the treatment of diabetes and obesity: a meta-analysis of randomized clinical trials [published 2019] |
|  | CRD42018081624 | A systematic review of clinical trials of homeopathy in urological disorders [published 07/2020] |
|  | CRD42017071609 | A systematic review of controlled trials of homeopathy in bronchial asthma [published 03/2019] |
|  | CRD42022367188 | Can homeopathy reduce antibiotic use while maintaining symptom control for acute otitis media? A systematic review and meta-analysis [published 10/2024] |

S.2 Literature search strategy

S.2.1 Concepts

S.2.1.1 Concept for databases

The strategy is designed for OVID (Medline). It will be adapted for the other databases.

Concept: Homeopathy

#1 Homeopathy/

#2 Materia medica

#3 "formularies, homeopathic" OR "pharmacopoeias, homeopathic"

#4 homeopath* OR homoeopath* OR homoop* OR omeop* OR homopath* OR potentis* OR potentiz* OR nosode*

Concept: Medical condition (e.g. insomnia)

#5 insomn*

#6 sleep*

#7 (exp "Sleep Initiation and Maintenance Disorders"/)

#8 ("Sleep Disorders"/)

#9 (Wakefulness/)

#10 dyssomn*

Concept: Study design (e.g. randomized controlled trials)

#11 exp Randomized Controlled Trial/

#12 "controlled clinical trial".pt.

#13 randomized.ab.

#14 placebo.ab.

#15 "Drug Therapy".fs.

#16 randomly.ab.

#17 trial.ab.

#18 groups.ab.

#19 exp animals/

#20 humans.sh.

#21 OR/1-4

#22 OR/5-10

#23 OR/11-18

#23 #19 NOT #20

#24 #21 AND #22 AND #23

#25 #24 NOT #23

S.2.1.2 Concept for websites and citation indexes

Websites and citation indexes will be searched the following ways:

AYUSH Research Portal

The medical system “homeopathy” will be combined with the “body system” (e.g. urinary system) and the resulting list of studies searched by hand.

Website of the Indian Journal for Research in Homeopathy

The interface will be screened for the keywords ‘controlled study’, ‘controlled trial’, ‘observational study’ and ‘observational trial’ in all fields.

Citation indexes

Web of Science (Thomson Reuters) will be searched using the terms “homeopathy” and terms corresponding to the medical condition (e.g. “insomnia”).

S.2.2 Demonstration of search strategies

MEDLINE (OVID)

homeopathy

#1 Homeopathy/ or Materia Medica/ or Pharmacopoeias, Homeopathic as Topic/ or Formularies, Homeopathic as Topic/ or (homeopath* or homoeopath* or homoop* or omeop* or homopath* or potentis* or potentiz* or "materia medica" or nosode*).tw.

study type

randomized controlled trials

#2 (exp Randomized Controlled Trial/ OR "controlled clinical trial".pt. OR randomized.ab. OR placebo.ab. OR "Drug Therapy".fs. OR randomly.ab. OR trial.ab. OR groups.ab.) NOT (exp animals/ NOT humans.sh.)

non-randomized studies on interventions

#3 (exp cohort studies/or exp epidemiologic studies/or exp clinical trial/or exp evaluation studies as topic/or exp statistics as topic/ or ((control and (group* or study)) or (time and factors) or program or survey* or ci or cohort or comparative stud* or evaluation studies or follow-up*).mp.) not ((animals/not humans/) or comment/or editorial/or exp review/or meta analysis/or consensus/or exp guideline/ or hi.fs. or case report.mp.)

condition (e.g. insomnia)

#4 exp "Sleep Initiation and Maintenance Disorders"/ or insomn*.ab,ti. OR sleep*.ab,ti. OR dyssomn*.ab,ti

#5 #1 AND #2 AND #4

#6 #1 AND #3 AND #4

EMBASE (OVID)

homeopathy

#1 homeopathic agent/ or homeopathy/ or materia medica/ or (homeopath* or homoeopath* or homoop* or omeop* or homopath* or potentis* or potentiz* or "materia medica" or nosode*).tw.

study type

randomized controlled trials

#2 (exp randomized controlled trial/ or controlled clinical trial/ or random$.ti,ab. or randomization/ or intermethod comparison/ or placebo.ti,ab. or (compare OR compared OR comparison).ti,ab. or ((evaluated OR evaluate OR evaluating OR assessed OR assess) AND (compare OR compared OR comparing OR comparison)).ab or (open adj label).ti,ab. or ((double OR single OR doubly OR singly) adj (blind OR blinded OR blindly)).ti,ab. or double blind procedure/ or parallel group$1.ti,ab. or (crossover OR cross over).ti,ab. or ((assign$ OR match OR matched OR allocation) adj5 (alternate OR group$1 OR intervention$1 OR patient$1 OR subject$1 OR participant$1)).ti,ab. or (assigned OR allocated).ti,ab. or (controlled adj7 (study OR design OR trial)).ti,ab. or (volunteer OR volunteers).ti,ab. or human experiment/ or trial.ti.) not ((random$ adj sampl$ adj7 ("cross section$" OR questionnaire$1 OR survey$ OR database$1)).ti,ab. NOT (comparative study/ OR controlled study/ OR randomi?ed controlled.ti,ab. OR randomly assigned.ti,ab.) or cross-sectional study/ NOT (exp randomized controlled trial/ OR controlled clinical trial/ OR controlled study/ OR randomi?ed controlled.ti,ab. OR control group$1.ti,ab.) or ((case adj control$) AND random$.ti,ab.) NOT randomi?ed controlled.ti,ab. or (systematic review.ti,ab. NOT (trial OR study).ti) or (non random$ NOT random$).ti,ab. or "random field$".ti,ab. or (random cluster adj3 sample$).ti,ab. or (review.ab. AND review.pt.) NOT trial.ti. or ("we searched".ab. AND (review.ti. OR review.pt.)) or "update review".ab. or (databases adj4 searched).ab. or (rat OR rats OR mouse OR mice OR swine OR porcine OR murine OR sheep OR lambs OR pigs OR piglets OR rabbit OR rabbits OR cat OR cats OR dog OR dogs OR cattle OR bovine OR monkey OR monkeys OR trout OR marmoset$1).ti. AND animal experiment/ or animal experiment/ NOT (human experiment/ OR human/))

non-randomized studies on interventions

#3 (exp cohort analysis/ or exp epidemiology/or exp clinical trial/or exp evaluation study/ or exp statistics/ or ((control and (group* or study)) or (time and factors) or program or survey* or ci or cohort or comparative stud* or evaluation studies or follow-up*).mp.) not ((animal/not human/) or editorial/or exp review/or meta analysis/or consensus/or exp practice guideline/ or case report.mp.)

condition (e.g. insomnia)

#4 exp sleep disorder/ or insomn*.ab,ti. OR sleep*.ab,ti. OR dyssomn*.ab,ti.

#5 #1 AND #2 AND #4

#6 #1 AND #3 AND #4

Cumulative Index to Nursing and Allied Health Literature (CINAHL) (Ebsco)

homeopathy

#1 ( (MH "Homeopathy") OR (MH "Homeopathic Agents+") ) OR TI ( (homeopath* or homoeopath* or homoop* or omeop* or homopath* or potentis* or potentiz* or "materia medica" or nosode*) ) OR AB ( (homeopath* or homoeopath* or homoop* or omeop* or homopath* or potentis* or potentiz* or "materia medica" or nosode*) )

condition (e.g. insomnia)

#3 (MH "Sleep Disorders+") OR TI ( insomn* OR sleep* OR dyssomn* ) OR AB ( insomn* OR sleep* OR dyssomn* )

#4 #1 AND #2 AND #3

Cochrane Library (search manager)

#1 (homeopath* or homoeopath* or homoop* or omeop* or homopath* or potentis* or potentiz* or "materia medica" or nosode*):ti,ab,kw (Word variations have been searched)

#2 (insomn* OR sleep* OR dyssomn*):ti,ab,kw (Word variations have been searched)

#3 #1 and #2

Allied and Complementary Medicine Database (AMED)

#1 (exp homeopathy/) OR (homeopath* or homoeopath* or homoop* or omeop* or homopath*).tw.

#2 (insomnia OR OR insomn* OR sleep OR dysomn*)

#3 #1 and #2

LILACS (Advanced search)

(homeopath* OR homoeopath*) AND (insomn* or dyssomnia or Sleep Disorders) AND (randomly OR randomised OR randomized OR RCT OR controlled trial OR double blind OR placebo OR cohort OR observational OR longitudinal) [title, abstract, subject]

Web of Science (Advanced search, Query preview)

((TI=(homeopath* OR homoeopath*)) OR (AB=(homeopath* OR homoeopathy)) OR AND (ALL=(insomnia OR sleep OR dysomn* OR insomn*))

PsycINFO

(via Ebsco)

#1 TI ( (homeopath* or homoeopath* or homoop* or omeop* or homopath* or potentis* or potentiz* or "materia medica" or nosode*) ) OR AB ( (homeopath* or homoeopath* or homoop* or omeop* or homopath* or potentis* or potentiz* or "materia medica" or nosode*) )

#2 DE "Sleep Wake Disorders" OR DE "Insomnia OR TI ( insomn* OR sleep* OR dyssomn* ) OR AB ( insomn* OR sleep* OR dyssomn* )

#3 #1 and #2

Web of Science

#1 ALL=(homeopath* or homoeopath* or homoop* or omeop* or homopath* or potentis* or potentiz* or "materia medica" or nosode*)

#2 ALL=(insomn* OR sleep* OR dyssomn* )

#3 #1 and #2

S.3 Pre-selection of health conditions

Table S.3: Complete list of patient populations for pre-selection by expert consensus panels

| ICD-10 | Indication | Study type | Studies | Studies (total) | patient populations | reason for exclusion |
| --- | --- | --- | --- | --- | --- | --- |
| A09.0 | Acute diarrhea, unspecified | RCT | 7 | 7 | acute diarrhea | NA |
| F51.0 | Insomnia | RCT | 8 | 10 | insomnia | NA |
| F51.0 | Insomnia | NRS | 2 |  |  |  |
| N95 | Menopausal and female climacteric states | RCT | 11 | 16 | female hormone regulation | NA |
| N94.4 | Primary dysmenorrhea |  |  |  |  |  |
| N94.3 | PMS | RCT | 5 |  |  |  |
| J06.9 | URTI | RCT | 8 | 33 | infectious diseases, antibiotic use |  |
| J06.9 | URTI | NRS | 7 |  |  |  |
| J22 | Respiratory tract infections | RCT | 5 |  |  |  |
| J22 | Respiratory tract infections | NRS | 5 |  |  |  |
| H66.9 | Otitis media | NRS | 4 |  |  |  |
| H66.9 | Otitis media | RCT | 4 |  |  |  |
| J30.4 | Allergic rhinitis | RCT | 7 | 22 | allergic rhinitis | NA |
| J30.1 | Seasonal allergic rhinitis | RCT | 12 |  |  |  |
| J30.1 | Seasonal allergic rhinitis | NRS | 3 |  |  |  |
| various | Arnica | NRSI | 4 | 28 | various | NA |
| various | Arnica | RCT | 24 |  |  |  |
| M54 | Back pain | RCT | 12 | 34 | musculosceletal pain | NA |
| M17.9 | Gonarthrosis | RCT | 7 |  |  |  |
| M79.7 | Pain in Fibromyalgia | RCT | 3 |  |  |  |
| M06.9 | Rheumatoid arthritis | NRS | 3 |  |  |  |
| M06.9 | Rheumatoid arthritis | RCT | 6 |  |  |  |
| M16.9 | Osteoarthritis of hip |  |  |  |  |  |
| M19.9 | Osteoarthritis, unspecified |  |  |  |  |  |
| M47.8 | Other spondylosis |  |  |  |  |  |
| M54 | dorsalgia |  |  |  |  |  |
| M17.9 | Gonarthrosis | NRS | 3 |  |  |  |
| J45.0 | Allergic asthma | RCT | 3 | 15 | asthma | NA |
| J45.9 | Asthma bronchiale, unspecified | NRS | 2 |  |  |  |
| J45.9 | Asthma bronchiale, unspecified | RCT | 10 |  |  |  |
| Z48.8 | Surgical follow up: all | RCT | 23 | 39 | wound healing/pain | NA |
| Z98.8 | Pain surgery | NRS | 2 |  |  |  |
| Z98.8 | Pain surgery | RCT | 11 |  |  |  |
| T14.9 | Pain from injuries | RCT | 3 |  |  |  |
| various | Arsenicum | RCT | 6 | 6 | various | NA |
| J11.1 | Influenza-like syndrome | RCT | 23 | 34 | influenza | NA |
| J11.1 | Influenza-like syndrome | NRS | 11 |  |  |  |
| I10 | Hypertension | RCT | 9 | 9 | hypertension | NA |
| J32.9 | Chronic sinusitis | RCT | 7 | 10 | chronic sinusitis | NA |
| J32.9 | Chronic sinusitis | NRS | 3 |  |  |  |
| E11.9 | Diabetes mellitus type 2 | RCT | 4 | 7 |  | NA |
| E11.9 | Diabetes mellitus type 2 | NRS | 3 |  |  |  |
| O75.9 | Complications during labour | RCT | 4 | 7 |  | NA |
| O75.9 | Complications during labour | NRS | 3 |  |  |  |
| C80.9 | Health related quality of life in Cancer | RCT | 2 | 4 | cancer | too few studies |
| C80.9 | Health related quality of life in Cancer | NRSI | 2 |  |  |  |
| K12.1 | Stomatitis | RCT | 5 |  |  | too few studies |
| G43.9 | Migraine | RCT | 4 |  |  | too few studies |
| E66.9 | Obesity | RCT | 5 |  |  | too few studies |
| F98.0 | Enuresis | RCT | 4 |  |  | too few studies |
| L20.9 | Atopic dermatitis | NRS | 3 |  |  | too few studies |
| F41.8 | Specific anxiety disorder | RCT | 6 |  |  | SR ongoing/published 2020 or later |
| F32.9 | Depressive episode | RCT | 4 |  |  | SR ongoing/published 2020 or later |
| F41.1 | Generalized axiety disorder | RCT | 4 |  |  | SR ongoing/published 2020 or later |
| T56.9 | Toxic effects of metals | RCT | 4 |  |  | too few studies |
| H81.9 | Vertigo | RCT | 3 | 6 |  | remedy of single company |
| various | Vertigoheel | RCT | 3 |  |  | remedy of single company |
| B07 | warts | RCT | 6 |  |  | too few studies |
| various | Galphima | RCT | 4 |  |  | remedy of single company |
| various | Gripp | RCT | 2 |  |  | remedy of single company |
| various | Zeel | RCT | 2 |  |  | remedy of single company |
| L70.0 | Acne vulgaris | RCT | 4 |  |  | too few studies |
| F90.0 | ADHD | RCT | 8 |  |  | SR ongoing/published 2020 or later |
| various | Engystol | RCT | 3 |  |  | remedy of single company |
| various | Engystol | NRSI | 3 |  |  | remedy of single company |
| various | Gripp | NRSI | 2 |  |  | remedy of single company |
| B23 | HIV | NRS | 2 | 4 |  | too few studies |
| B23 | HIV | RCT | 2 |  |  | too few studies |
| various | Hypericum/Arnica | RCT | 3 |  |  | too few studies |
| M62.6 | Muscle strain | RCT | 3 |  |  | too few studies |
| B48.7 | Opportunistic oral thrush | RCT | 2 | 4 |  | too few studies |
| B48.7 | Opportunistic oral thrush | NRS | 2 |  |  | too few studies |
| various | Oscillococcinum | RCT | 7 | 9 |  | remedy of single company |
| various | Oscillococcinum | NRSI | 2 |  |  | remedy of single company |
| K05.3 | Periodontosis | RCT | 3 |  |  | too few studies |
| C80.9 | Survival in cancer | NRS | 3 |  |  | too few studies |
| various | Traumeel S | RCT | 7 |  |  | remedy of single company |
| various | Traumeel S | NRSI | 8 |  |  | remedy of single company |
| H81.9 | Vertigo | NRS | 6 |  |  | remedy of single company |
| various | Vertigoheel | NRSI | 5 |  |  | remedy of single company |

S.4 iCAT_SR details

Table S.4: iCAT_SR dimensions and their relevance for studies on homeopathy

| Dimension | | included | Comment | Adapted instructions |
| --- | --- | --- | --- | --- |
| core dimensions | | | |  |
| 1 | Active components included in the intervention, in relation to the comparison | yes | Active components of the intervention can be (A) Single homeopathic medicinal products (HPs), (B) the patient-therapist interaction, (C) co-interventions. In the cases of A and B, it may not be possible, however, to meaningfully separate the active components from each other. When homeopathy is administered with co-therapies, the intervention may range from a single active element to an integrated package or vary within a study. | Define the number of active components in the intervention.  Details: Components can be defined as “the essential functions or principles, associated actors and processes and intervention activities that are judged necessary to produce desired outcomes” ([doi: 10.1186/s12889-021-11244-3](sps:urlprefix::http)). Example: Rate A for a complex HP, B for multiple HPs or individualized therapy (single HP plus patient-therapist interaction, and C for any type of homeopathy plus add-on. Don’t rate standard care as a component. See domain 1 of iCAT_SR.  A: 1 component  B: bundle of components  C: multiple components |
| 2 | Behaviours or actions of intervention recipients or participants to which the intervention is directed | no | Although it is possible that studies combine the intake of HP with co-therapies aimed at behavioural changes (e.g dietary advice), we exclude studies in which the effect attributable to the HP cannot be estimated. Eligible studies would therefore need to compare HP plus behavioural changes to behavioural changes alone and not to another control. In most studies, the intervention is directed at one action only. | NA |
| 3 | Organisational levels and categories targeted by the intervention | no | The intervention is always directed at individuals. | NA |
| 4 | The degree of tailoring intended or flexibility permitted across sites or individuals in applying or implementing the intervention | yes | This dimension corresponds to the degree of individualization of homeopathy and ranges from "minimal" (e.g. Vertigoheel yes/no) to "highly tailored" (fully individualized without restriction). | Define the degree of tailoring intended across individuals in applying the intervention.  Details: The degree of tailoring a homeopathic intervention is related to its individualization. Thus, complex, single non-isopathic, isopathic, restricted individualized (limited in the selection of homeopathic remedies), and unrestricted individualized homeopathy (not limited in the selection of remedies) are on the scale from “not individualized” to “fully individualized”. The degree of tailoring can be reflected in the flexibility of the intervention, but it can also be an inherent part of the study design (e.g., when a specific patient population receives a preparation that has been preselected for them through homeopathic individualization). See domain 4 of iCAT_SR.  A: clinical, complex/multi-constituents remedies  B: clinical, single-constituents (one remedy only)  C: clinical, isopathy  D: partly individualized (selection from predefined list or restricted change during time)  E: fully individualized |
| 5 | The level of skill required by those delivering the intervention in order to meet the intervention objectives | no | The required level of skill depends on the degree of individualization and is thus linked to dimension 4. | NA |
| 6 | The level of skill required for the targeted behaviour when entering the included studies by those receiving the intervention (consumers, professionals, planners), in order to meet the intervention objectives | no | For the majority of studies, no specialised skills are needed by the patients of homeopathic therapies (see dimension 2). | NA |
| optional dimensions | | | |  |
| 7 | The degree of interaction between intervention components, including the independence / interdependance of intervention components | no | Intervention components are the HPs, the patient-therapist interaction and co-interventions. Their degree of dependence may be related to the level of individualization and is therefore linked to dimension 4. | NA |
| 8 | The degree to which the effects of the intervention are dependent on the context or setting in which it is implemented | no | Context factors can be person-related (see dimension 9) or external such as the physical and social environment if they are perceived by the patient or the provider and considered important. For instance, factors such as the setting (primary care, hospital) may modulate expectations and, in turn, patient-reported outcomes. However, this domain cannot be assessed based on the information commonly provided by the studies. | NA |
| 9 | The degree to which the effects of the intervention are changed by recipient or provider factors | no | Characteristics of the patient, e.g. level of chronicity of the disease, co-morbidities, etc. are expected to modify the effects. Many effect outcomes in homeopathic studies are patient-reported. It is possible that the perceived effects, but also the patient-therapist interaction alter depending on socioeconomic factors, including income, education, health beliefs, disease history, treatment expectations, literacy levels. A minimally individualized approach may be less modfied by individual factors. The impact of the provider depends on the degree of individualization and include his/her education (e.g. homeopathic school), past experience and homeopathic tools (repertoirs, text books, software). | NA |
| 10 | The nature of the causal pathway between the intervention and the outcome it is intended to effect | no | iCAT_SR refers to pathways that involve human actions (such as behaviours) or actions within organisations rather than biological pathways. Thus, this dimension is not relevant to assess the intervention complexity of homeopathy. | NA |

S.5 Data extraction form examples

Table S5: Characteristics of included studies (example)

| Item | Instruction |
| --- | --- |
| Identification |  |
| Study ID | 1st author’s last name and year of publication (e.g. Smith_1989) |
| Reference | Include all reports, registrations, protocols. |
| Country | List countries. |
| Source of funding | List source of funding (or not specified). |
| Sponsor category | Corporate, public, no funding, no information |
| Conflict of interest | List declared interests (or none declared). |
| Enrollment period | List enrolment dates (e.g. Jan 2023 to Feb 2024). |
| Lenght of treatment | List treatment duration in days (convert weeks, months, years to 7, 30, 360 days). |
| Lenght of follow-up | List follow-up in days (convert weeks, months, years to 7, 30, 360 days). |
| Author's contact details |  |
| Name | Provide last name, first name (e.g. Smith, Jane). |
| Institution | Enter name of institution of 1st author (e.g. Harvard TH Chan School of Public Health). |
| Email | Enter email-address of corresponding author. |
| Method details |  |
| Design | Enter study design (e.g. RCT, NRSI). |
| Single or multicentre study | Enter whether the study is single or multi-center (single, multi center, NA). |
| Groups | Enter the number of treatment arms. |
| Statistical analysis | Provide details (e.g. descriptive, ANOVA). |
| Setting | List setting (e.g., university hospital, nursing home, community). |
| Provider | Provide details (e.g. trained homeopath with a minimum of 3 years experience). |
| Population |  |
| Population details | Provide brief details (e.g. children with acute otitis media). |
| Inclusion criteria | List all inclusion criteria. |
| Exclusion criteria | List all exclusion criteria. |
| Total sample size (NRSI, RCT) | Enter the total number of participants enrolled (e.g. 233). |
| Gender (number of females) | Enter the number of females (e.g. 123). |
| Mean age | Enter the mean age in years (e.g. 35,7). |
| Co-morbidities | List all co-morbidities. |
| Interventions |  |
| Homeopathy | Describe the homeopathic intervention (e.g. Individualized homeopathy with free choice of medicinal product, potency used C30). |
| Control | Describe the control intervention (e.g. placebo). |
| add-on | Was the intervention used as add-on therapy (yes, no, unknown)? |
| Co-interventions | List all co-interventions. |
| Lenght of treatment | List treatment duration in days (convert weeks, months, years to 7, 30, 360 days). |
| Lenght of follow-up | List follow-up in days (convert weeks, months, years to 7, 30, 360 days). |
| iCAT_SR dimension 1 | Active components included in the intervention, in relation to the comparison. |
| iCAT_SR dimension 4 | The degree of tailoring intended or flexibility permitted across sites or individuals in applying or implementing the intervention. |
| Statistical analysis |  |
| Population details | Provide details (e.g. intention-to-treat). |
| Population included | Provide the number of patients included per treatment arm. |
| Missing data | Provide details (e.g. Change to baseline SD missing, imputation according to protocol). |
| Outcomes | Describe outcomes (primary/secondary, timing, measurement scale). |

Abbreviations

Abbreviations

ANOVA: analysis of variance, NRSI: non-randomized studies of intervention, RCT: randomized controlled trials, SD: standard deviation

Outcome measures (example)

| Study_ID | Population | homeopathy | Control | Outcome | Scale | Scale_range | Timing (months) | Measure details | HO_Mean | HO_SD | HO_n | CO_Mean | CO_SD | CO_n | direction |
| --- | --- | --- | --- | --- | --- | --- | --- | --- | --- | --- | --- | --- | --- | --- | --- |
| Witt 2009 | children with mild to moderate atopic eczema | individualized HO | conventional medicine | symptom severity | SCORAD (Scoring Atopic Dermatitis) | [0;103] | 6 | mean change from baseline | -8,80 | 21,46 | 48 | -4,60 | 23,99 | 87 | favors HO |
|  |  |  |  | symptom severity | SCORAD (Scoring Atopic Dermatitis) | [0;103] | 12 | mean change from baseline |  |  |  |  |  |  |  |
|  |  |  |  | number of medications (12 months) | NA | NA | 12 |  |  |  |  |  |  |  |  |
|  |  |  |  | outcome 4 |  |  |  |  |  |  |  |  |  |  |  |
|  |  |  |  | outcome 5 |  |  |  |  |  |  |  |  |  |  |  |
|  |  |  |  | outcome 6 |  |  |  |  |  |  |  |  |  |  |  |
|  |  |  |  | outcome 7 |  |  |  |  |  |  |  |  |  |  |  |

Abbreviations

Abbreviations

CO: control, HO: homeopathy, NA: not available, SD: standard deviation

S.6 PRISMA-P Checklist

Table S6: PRISMA-P Checklist

| Section and topic | Item No | Checklist item | Location in protocol |
| --- | --- | --- | --- |
| ADMINISTRATIVE INFORMATION | | |  |
| Title: |  |  |  |
| Identification | 1a | Identify the report as a protocol of a systematic review | Title |
| Update | 1b | If the protocol is for an update of a previous systematic review, identify as such | NA |
| Registration | 2 | If registered, provide the name of the registry (such as PROSPERO) and registration number | NA |
| Authors: |  |  |  |
| Contact | 3a | Provide name, institutional affiliation, e-mail address of all protocol authors; provide physical mailing address of corresponding author | Title |
| Contributions | 3b | Describe contributions of protocol authors and identify the guarantor of the review | supporting info |
| Amendments | 4 | If the protocol represents an amendment of a previously completed or published protocol, identify as such and list changes; otherwise, state plan for documenting important protocol amendments | NA |
| Support: |  |  |  |
| Sources | 5a | Indicate sources of financial or other support for the review | supporting info |
| Sponsor | 5b | Provide name for the review funder and/or sponsor | supporting info |
| Role of sponsor or funder | 5c | Describe roles of funder(s), sponsor(s), and/or institution(s), if any, in developing the protocol | supporting info |
| INTRODUCTION | | |  |
| Rationale | 6 | Describe the rationale for the review in the context of what is already known | chapter 5 |
| Objectives | 7 | Provide an explicit statement of the question(s) the review will address with reference to participants, interventions, comparators, and outcomes (PICO) | chapter 5 |
| METHODS | | |  |
| Eligibility criteria | 8 | Specify the study characteristics (such as PICO, study design, setting, time frame) and report characteristics (such as years considered, language, publication status) to be used as criteria for eligibility for the review | chapter 6.2 |
| Information sources | 9 | Describe all intended information sources (such as electronic databases, contact with study authors, trial registers or other grey literature sources) with planned dates of coverage | chapter 6.3 |
| Search strategy | 10 | Present draft of search strategy to be used for at least one electronic database, including planned limits, such that it could be repeated | chapter S.2 |
| Study records: |  |  |  |
| Data management | 11a | Describe the mechanism(s) that will be used to manage records and data throughout the review | chapter 6.5 |
| Selection process | 11b | State the process that will be used for selecting studies (such as two independent reviewers) through each phase of the review (that is, screening, eligibility and inclusion in meta-analysis) | chapter 6.4 |
| Data collection process | 11c | Describe planned method of extracting data from reports (such as piloting forms, done independently, in duplicate), any processes for obtaining and confirming data from investigators | chapter 6.5 |
| Data items | 12 | List and define all variables for which data will be sought (such as PICO items, funding sources), any pre-planned data assumptions and simplifications | chapter 6.5, S.5 |
| Outcomes and prioritization | 13 | List and define all outcomes for which data will be sought, including prioritization of main and additional outcomes, with rationale | chapter 6.9 |
| Risk of bias in individual studies | 14 | Describe anticipated methods for assessing risk of bias of individual studies, including whether this will be done at the outcome or study level, or both; state how this information will be used in data synthesis | chapter 6.6 |
| Data synthesis | 15a | Describe criteria under which study data will be quantitatively synthesised | chapter 6.12 |
|  | 15b | If data are appropriate for quantitative synthesis, describe planned summary measures, methods of handling data and methods of combining data from studies, including any planned exploration of consistency (such as I2, Kendall’s τ) | chapter 6.11 |
|  | 15c | Describe any proposed additional analyses (such as sensitivity or subgroup analyses, meta-regression) | chapter 6.12 |
|  | 15d | If quantitative synthesis is not appropriate, describe the type of summary planned | chapter 6.12 |
| Meta-bias(es) | 16 | Specify any planned assessment of meta-bias(es) (such as publication bias across studies, selective reporting within studies) | chapter 6.14 |
| Confidence in cumulative evidence | 17 | Describe how the strength of the body of evidence will be assessed (such as GRADE) | chapter 6.17 |

S.7 Advisory Patient and Public Involvement Approach

[Purpose](sps:id::sec1) and scope

This appendix describes a standardized approach for advisory Patient and Public Involvement (PPI) applied across the series of systematic reviews. It complements the main protocol by outlining how patient perspectives are incorporated to enhance relevance, transparency, and accessibility, particularly with respect to outcome selection, interpretation of findings, and the development of plain-language summaries.

[Nature](sps:id::sec2) of involvement

Patient involvement is strictly advisory. Patient advisors provide feedback on:

• the relevance and clarity of review topics and objectives at the protocol stage;

• the appropriateness and patient relevance of selected outcomes;

• the clarity and comprehensibility of plain-language summaries and interpretations of findings.

Patient advisors do not participate as research subjects, and no experimental procedures or data collection for research purposes takes place.

[Identification](sps:id::sec3) and selection of patient advisors

Patient advisors are nominated by the European Federation of Homeopathic Patients’ Associations (EFHPA) for each indication area addressed within the review series. Typically, five or more advisors are involved per indication area.

Advisors are expected to:

• have experienced the indication in question,

• be able to review short written materials (approximately 1–2 pages),

• participate in one or two brief online consultations or written feedback rounds.

Efforts are made to achieve a balanced representation with respect to age, gender, and relevant patient experience.

[Methods](sps:id::sec4) of engagement

PPI activities are conducted using:

• standardized online questionnaires with open and closed questions, and/or

• written feedback on plain-language materials.

No personal health data are requested or collected.

[Development](sps:id::sec5) and review of plain-language materials

Plain-language summaries are developed for both the protocol and the final systematic review manuscript.

Standardized AI prompts are used to generate an initial draft lay summary for each document. The lead review author then develops the lay summaries on the basis of these AI-generated drafts, in accordance with the Cochrane Plain Language Summary template (Pitcher et al., 2022).

The specific ChatGPT model and prompt version used are documented in the PPI log. An experienced patient representative with expertise in developing plain-language health summaries reviews the texts prepared by the lead review author for clarity, balance, and patient comprehensibility.

[Documentation,](sps:id::sec6) reporting, and transparency

In each systematic review a concise PPI log is maintained. The lead review author is responsible for documenting:

• the timing of PPI consultations,

• the number of patient advisors involved (anonymised),

• the main themes of the feedback received, and

• how the feedback was addressed.

EFHPA manages communication with patient advisors and contact information but does not document feedback content or influence review decisions.

PPI activities and their impact are reported in accordance with the GRIPP2 reporting guideline [[54]](SPS:refid::bib54) and summarised in the “Consumer Involvement” sections of the published reviews. Patient advisors are acknowledged in publications; co-authorship may be offered if contributions meet ICMJE criteria.

[Integration](sps:id::sec7) of patient feedback

Patient advisors’ feedback is analysed by the lead author using a concise qualitative thematic approach. Feedback is summarised and grouped into thematic categories (e.g. relevance of outcomes, clarity of materials, interpretation of findings).

Integration decisions are documented transparently using a three-level code: feedback fully integrated; feedback partially integrated (with justification); feedback not integrated (with justification, e.g. methodological constraints).

A second team member conducts a brief selective review to ensure transparency and plausibility of the integration decisions.

[Ethics,](sps:id::sec8) data protection, and confidentiality

The PPI activities constitute consultation rather than research purposes. No sensitive personal data are collected, and no ethics approval is required. Advisors may withdraw at any time. All feedback is anonymised and reported in aggregated form.

[References](sps:id::sec9)

Pitcher, N., Mitchell, D., & Hughes, C. (2022). Template and guidance for writing a Cochrane Plain language summary. Retrieved 17.12.2025, from [https://www.cochrane.org/fr/authors/handbooks-and-manuals/handbook/current/guidance-writing-cochrane-plain-language-summary.pdf](sps:urlprefix::https)

Staniszewska, S., Brett, J., Simera, I., Seers, K., Mockford, C., Goodlad, S., Altman, D. G., Moher, D., Barber, R., Denegri, S., Entwistle, A., Littlejohns, P., Morris, C., Suleman, R., Thomas, V., & Tysall, C. (2017). GRIPP2 reporting checklists: tools to improve reporting of patient and public involvement in research. BMJ, 358, j3453. [https://doi.org/10.1136/bmj.j3453](sps:urlprefix::https)
